# Coordinated Virulence in *Acinetobacter baumannii:* Mechanisms, Integration and Therapeutic Opportunities

**DOI:** 10.3390/pathogens15101092

**Published:** 2026-10-20

**Authors:** Luis A. Ramos, Peter T. Brancato, William F. Penwell, Alfred C. Gitu, Jennifer T. Grier, Luis A. Actis, Aria Eshraghi, Steven E. Fiester

**Affiliations:** 1 Department of Biological Sciences, Florida Gulf Coast University, Fort Myers, FL 33965, USA; lramoshernandez@fgcu.edu (L.A.R.); ptbrancato0527@eagle.fgcu.edu (P.T.B.); 2 Department of Biology and Marine Science, Jacksonville University, Jacksonville, FL 32211, USA; 3 Family Medicine Residency Program at Lee Health, College of Medicine, Florida State University, Fort Myers, FL 33901, USA; 4 Academic Medicine Enterprise, Lee Health, Fort Myers, FL 33912, USA; 5 Department of Biomedical Sciences, University of South Carolina School of Medicine Greenville, Greenville, SC 29605, USA; 6 Department of Pathology, Prisma Health Upstate, Greenville, SC 29605, USA; 7 Department of Microbiology, Miami University, Oxford, OH 45056, USA; actisla@miamioh.edu; 8 Department of Infectious Diseases and Immunology, University of Florida, Gainesville, FL 32608, USA; ariaeshraghi@ufl.edu; 9 Internal Medicine Residency Program at Lee Health, College of Medicine, Florida State University, Cape Coral, FL 33990, USA; 10 Department of Chemistry, Furman University, Greenville, SC 29613, USA; 11 School of Health Research, Clemson University, Clemson, SC 29634, USA

**Keywords:** *Acinetobacter*, virulence factor, biofilm, outer membrane protein, capsule, phospholipase, pili

## Abstract

*Acinetobacter baumannii* has emerged as a significant multi-drug-resistant (MDR) pathogen, particularly in healthcare environments, due to its large arsenal of virulence factors. This review organizes these virulence factors into three groups based primarily on location and functionality: (1) membrane-associated virulence factors, (2) outer envelope components and secreted virulence factors and (3) pili and fimbriae. Together, these factors drive motility, biofilm formation, abiotic and biotic surface adherence, cell invasion, immune evasion, and antimicrobial resistance, which all complicate infection management. Elucidation of the individual and coordinated mechanisms of bacterial virulence factors is essential for identifying targets that can be exploited by novel therapies. This review, therefore, synthesizes the literature to date describing *A. baumannii* virulence factors as well as their individual and concerted functionalities to provide groundwork for future studies that will hopefully aid in the discovery of novel therapeutics.

## 1. Introduction

*Acinetobacter baumannii* is an opportunistic Gram-negative pathogen responsible for an ever-increasing number of healthcare-associated infections, including bacteremia, meningitis, pneumonia, skin and soft tissue infections, and urinary tract infections. These infections commonly affect vulnerable populations with severe trauma, immunosuppression, burns, mechanical ventilation or catheterization [1,2]. In the United States alone, *A. baumannii* causes an estimated 7000 infections and 500 deaths annually [3]. Both the World Health Organization (2017) and Centers for Disease Control and Prevention (2019) have designated multi-drug-resistant (MDR) *A. baumannii* as a high-priority pathogen and urgent threat, respectively, reflecting the clinical significance of this pathogen and underscoring the urgent need for development of new therapeutic strategies to treat *A. baumannii* infections [4].

A key contributor to the threat of severe or drug-resistant infection by *A. baumannii* is the bacterium’s capacity to persist in both clinical environments and the host, an ability that is driven by a diverse and likely functionally coordinated repertoire of virulence factors that promote pathogenicity. Translocase proteins such as SecA direct the location of a variety of virulence factors [5,6]. While outer membrane proteins (OMPs) including OmpA and CarO support pathogenicity by contributing to antibiotic resistance as well as cell adhesion, invasion and apoptosis, in part through inclusion in outer membrane vesicles (OMVs) [7,8,9]. In addition, secreted and surface-associated factors such as phospholipases and lipooligosaccharide (LOS) promote survival mechanisms including serum resistance, biofilm development and immune evasion, while the capsular polysaccharide enhances survival in vivo by further protecting against host defenses [10,11,12,13]. Surface structures such as pili and fimbriae facilitate attachment to biotic and abiotic surfaces, promote biofilm formation and enable processes such as twitching motility and horizontal gene transfer [14,15].

Despite extensive characterization of individual virulence determinants, the integrated mechanisms by which these factors are regulated and function together during infection have yet to be elucidated. This lack of synthesis presents significant barriers to the development of urgently needed therapies to combat the growing prevalence of MDR *A. baumannii* infections [16]. The following review provides a comprehensive overview of the known mechanisms and functional roles of *A. baumannii* virulence factors, with emphasis on their coordination and potential as therapeutic targets.

## 2. Membrane-Associated Virulence Factors

**Figure 1:**
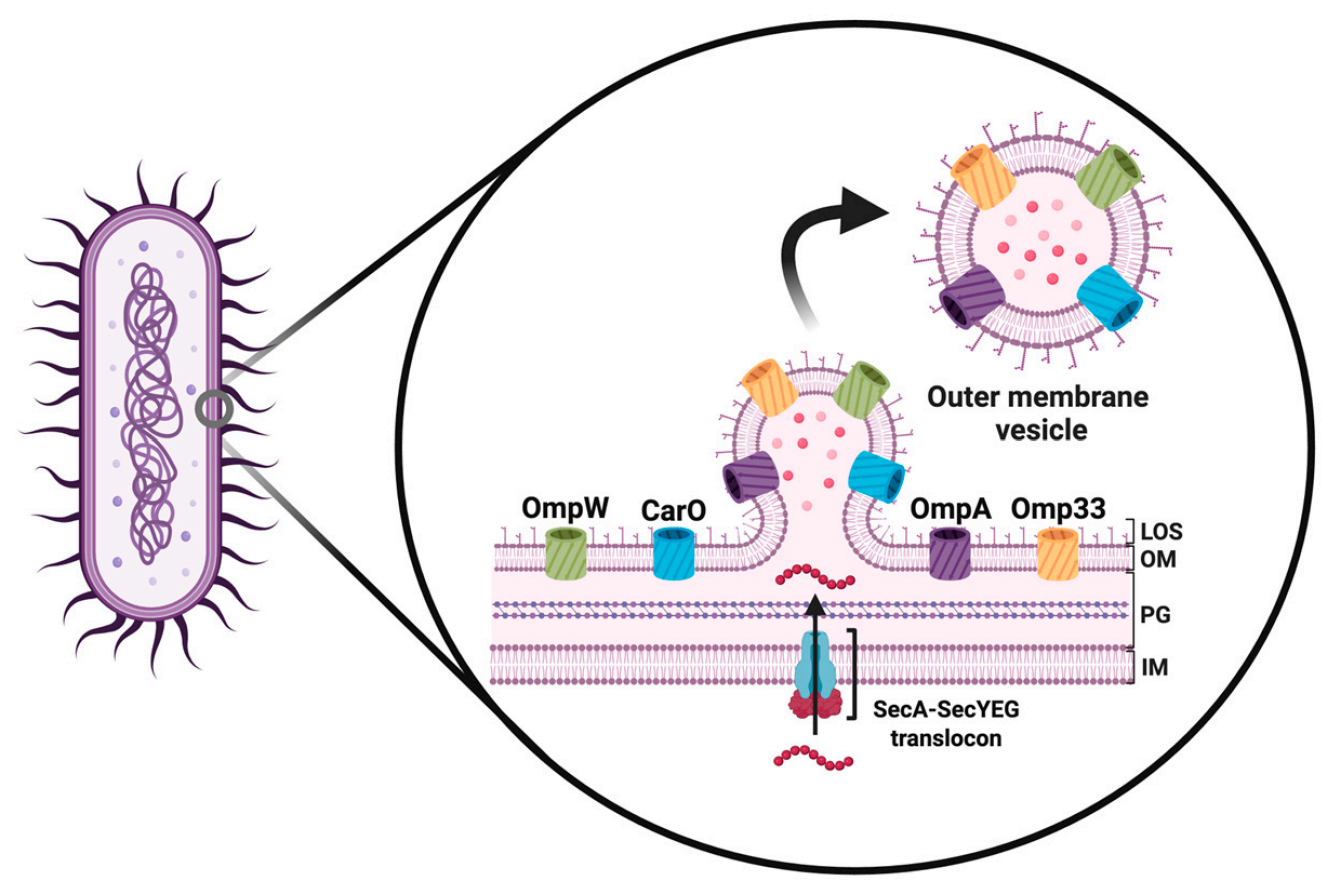
Schematic representation of major membrane-associated virulence factors of *A. baumannii.* Outer membrane protein A (OmpA), carbapenem-associated outer membrane protein (CarO), outer membrane protein 33-36 (Omp33) outer membrane protein W (OmpW) and the SecA translocase protein export pathway are highlighted within the *A. baumannii* cell envelope alongside a budding outer membrane vesicle (OMV). Created in BioRender. Brancato, P. (2026) https://BioRender.com/ry8hmse (accessed on 20 September 2026).

Membrane-associated proteins form a pivotal point of interface between *A. baumannii* and its environment, contributing to both structural integrity and virulence (Figure 1). These proteins are embedded within or associated with the inner and outer membranes, where they mediate key interactions with host tissues, antimicrobial agents, and environmental stressors. Most outer membrane proteins (OMPs) adopt *β*-barrel structures composed of 8–26 strands, featuring extended extracellular loops and short periplasmic regions that together enhance membrane stability under harsh conditions [17]. Functionally, OMPs such as OmpA, CarO and Omp33 contribute to virulence-associated processes, including cell adhesion, apoptosis of host cells, antibiotic resistance and amino acid uptake [9,18,19,20,21,22]. These surface-exposed proteins do not act in isolation but instead form a coordinated network that functionally links membrane structure to host interaction and survival, with specific examples of this coordination detailed in Section 5. In addition to outer membrane components, inner membrane systems such as the SecA-dependent secretion pathway play a critical supporting role by enabling the proper localization of a variety of virulence factors, including OMPs and components of iron acquisition systems [23]. Extending beyond the cell surface, OMVs further expand the functional reach of membrane-associated factors by delivering virulence determinants and resistance elements to host cells and neighboring bacteria. These vesicles have been implicated in adhesion, antibiotic resistance and modulation of host immune responses [8,24,25]. These membrane-associated systems highlight how *A. baumannii* leverages its membrane architecture not only as a protective barrier but also as a dynamic platform for virulence, communication and environmental adaptation.

### 2.1. SecA: A Gatekeeper of Virulence Coordination and Protein Export

The SecA translocase functions as a central coordinator of virulence in *A. baumannii* by governing the export of proteins required for host adaptation and antimicrobial resistance [5]. Rather than serving solely as a housekeeping component of the general secretory pathway, SecA appears to act as a functional bottleneck through which multiple virulence pathways converge, linking nutrient acquisition, envelope composition and resistance mechanisms. SecA mediates ATP-dependent translocation of unfolded proteins through the SecYEG translocon into the periplasm, where they are subsequently incorporated into the outer membrane or secreted [5,26,27]. While this function is broadly conserved, specific evidence in *A. baumannii* highlights a more specialized role for SecA in deploying virulence-associated outer membrane proteins and components associated with nutrient acquisition [23].

Studies in *Escherichia coli* show that SecA can exist in both soluble and membrane-bound forms, switching between them through ATP-dependent interactions with the SecYEG channel and membrane lipids [6,28,29]. However, a large portion of SecA does remain stably associated with the cytoplasmic membrane and does not participate in this cycling [30]. Microscopy studies have shown that SecA can form a ring-like pore that interacts with membrane phospholipids and becomes active when bound to the chaperone SecB [31,32,33]. Though not as specific and efficient as the SecA-SecYEG channel in the inner membrane, SecA alone can form protein-conducting channels and promote translocation into liposomes [34].

Disruption of SecA impairs proper localization of BauA, a receptor required for uptake of the iron-scavenging siderophore acinetobactin (ferric acinetobactin), resulting in reduced growth of *A. baumannii* under iron-limiting conditions and attenuated virulence in infection models. For example, the SecA-SecYEG translocon plays a crucial role in the ability of *A. baumannii* ATCC 19606^T^ to process and export BauA [23,35,36,37], with a transposon mutant of *secA* showing significantly reduced growth when cultured in iron-restrictive media. This *secA* mutant was able to produce BauB and BauE but lacked BauA in the outer membrane and thus was unable to scavenge iron using acinetobactin. Compared to wild type *A. baumannii*, *secA*-deficient *A. baumannii* displayed significantly attenuated virulence when tested using *Galleria mellonella* larvae as an experimental infection model [23]. These findings illustrate a key principle: SecA-mediated protein secretion capacity directly correlates with the bacterium’s ability to compete for essential nutrients within the host.

In addition to nutrient acquisition, SecA-dependent trafficking contributes to antibiotic resistance. Carbapenem-hydrolyzing enzymes, including oxacillinase (OXA)-type *β*-lactamases, require periplasmic localization, and, in some cases, incorporation into OMVs for optimal activity [38]. Carbapenem-resistant bacteria can “shelter” susceptible *A. baumannii* in polymicrobial infections, allowing them to survive antibiotic treatment increasing overall pathogenicity [39]. This suggests that SecA contributes not only to cell-intrinsic resistance, but also to community-level antibiotic tolerance, an underappreciated feature of *A. baumannii* infections. Pharmacological inhibition of the SecA pathway with rose bengal disrupts this bystander protection effect by blocking the periplasmic translocation of carbapenemases such as OXA-58, which is necessary for secretion via outer membrane vesicles [40]. When rose bengal is combined with imipenem in *A. baumannii*–*E. coli* co-culture, survival of the carbapenem-resistant *A. baumannii* strain is reduced, while the susceptible *E. coli* population is eliminated, indicating loss of this protective “sheltering” effect. Further studies show that low concentrations of rose bengal (<12.5 mg/L) decrease translocation of additional carbapenemases, including OXA-23 and OXA-83 [41]. However, higher concentrations of rose bengal (25 mg/L) produce mixed effects, with increased cytoplasmic expression of some enzymes (OXA-72 and OXA-23), while cytoplasmic OXA-83 expression decreased, suggesting a more complex or compensatory response [41].

SecA protein translocation mechanisms contribute significantly to the ability of *A. baumannii* to cause infections due to their roles in the transport of ferric acinetobactin and carbapenemases to effectively scavenge iron and resist antibiotics, respectively. The transport of these proteins is highly favorable towards or dependent upon the usage of SecA for translocation, and this SecA-facilitated transport favors both monomicrobial and polymicrobial infections [39]. The dual role of SecA in nutrient acquisition and resistance highlights its function as a functional hub during infection, integrating metabolic fitness with defense against antimicrobial stress. In the host environment where iron limitation and antibiotic exposure often occur simultaneously, this coordination may provide a selective advantage by linking environmental sensing with efficient deployment of adaptive factors. The centrality of SecA and lack of a mammalian counterpart has made it an attractive antimicrobial target [42]. Application against Gram-negative organisms has proven difficult for several reasons. Several classes of SecA inhibitors that are significantly effective against Gram-positive bacteria had markedly reduced activity against several Gram-negative species, including *A. baumannii* [43]. This was attributed to the permeation barrier of the additional outer membrane, since using membrane permeabilizers led to markedly increased sensitivity, even in MDR strains with elevated efflux activity [43]. Additionally, the large amount of Sec-dependent export may lead to difficulty when determining antimicrobial dosage. As previously seen in experiments with rose bengal, the effect of the compound changes direction across a two-fold concentration, showing the unpredictability when targeting this central gatekeeper [40,41].

### 2.2. OmpA: A Multifunctional Hub Driving Host Interaction, Immune Evasion and Persistence

The outer membrane protein A (OmpA) also functions as a central virulence hub in *A. baumannii*, integrating functions of structural integrity with host interaction and immune evasion. Structurally, OmpA consists of an N-terminal 8-stranded *β*-barrel embedded in the outer membrane and a C-terminal periplasmic domain that interacts with the cell wall [44,45,46]. While this architecture is typical of porins, OmpA is more than simply a passive transporter, instead it acts as a multifunctional effector that links adhesion, invasion, apoptosis, and serum and drug resistance phenotypes [7,17,18,47,48,49].

A defining feature of OmpA is its ability to directly manipulate host cell signaling to promote invasion and cell death. Both in vitro and in vivo models have demonstrated that the interaction of OmpA with toll-like receptor 2 (TLR2) initiates signaling through NF-κB, leading to pro-inflammatory cytokine production, cytoskeletal rearrangement and internalization of E-cadherin, collectively weakening epithelial barrier integrity and facilitating bacterial entry into lung epithelial cells [50]. Once internalized, OmpA further contributes to host damage through multiple mechanisms. Proteolytic fragments of OmpA translocate to the host nucleus and exhibit endonuclease activity that induces irreversible DNA damage and cell death, while OmpA also localizes to host mitochondria, triggering apoptosis via cytochrome c release, caspase activation and apoptosis-inducing factor (AIF) release [17,51]. These findings indicate that OmpA is not merely an adhesin, but rather a direct mediator of host cell injury, capable of engaging both nuclear and mitochondrial pathways to drive cell death. In parallel, OmpA enhances bacterial survival within the host by promoting immune evasion. Binding of complement regulatory protein Factor H by OmpA and other OMPs enables *A. baumannii* to resist complement-mediated killing, increasing survival in human serum [49]. Loss of OmpA significantly reduces dissemination from the lungs in vivo, likely reflecting combined defects in host cell invasion and complement resistance [48,49]. These observations suggest that OmpA coordinates entry into host tissues and persistence within the bloodstream, highlighting its dual role in invasion and systemic infection.

Beyond host interaction, OmpA contributes to phenotypes that support long-term bacterial persistence, including antibiotic resistance and biofilm formation. Mutants lacking OmpA display increased susceptibility to multiple antibiotics, suggesting that OmpA influences outer membrane permeability or stability in ways that modulate drug access [18,52]. Additionally, the restoration of biofilm formation following *ompA* mutant complementation indicates a key role for OmpA in surface-associated growth and community behavior [53]. OmpA enrichment in outer membrane vesicles (OMVs), and its role in virulence factor delivery are detailed in Section 2.3 [7,47]. Together, these findings position OmpA as a multiscale virulence determinant, operating at the level of individual cells, host tissues and bacterial communities.

The high conservation of OmpA (~99%) across *Acinetobacter* species and its strong immunogenicity make it an attractive candidate for vaccine development [54]. Several studies have demonstrated that OmpA-based immunization strategies confer significant protection in murine infection models, supporting its potential as a broadly effective vaccination antigen [54,55,56,57,58]. Its multifunctionality however raises important questions about whether targeting OmpA alone will sufficiently disrupt virulence or whether compensatory pathways may limit therapeutic efficacy. Factor H recruitment, relevant to serum resistance, is mediated by OmpA as well as other outer membrane proteins, so neutralizing OmpA alone would not be expected to completely abolish resistance [49]. Additionally, since a substantial amount of its cytotoxic activity is mediated by OMVs, an agent targeting surface-exposed OmpA may not completely neutralize the vesicle-associated virulence [8].

Despite extensive study, key aspects of OmpA biology remain unresolved. It is unclear how its diverse functions, such as adhesion, immune modulation, apoptosis induction and OMV biogenesis, are coordinated or regulated during infection. Additionally, the relative contribution of each function to disease progression as a factor of infection site and host environment is not yet understood. Addressing these gaps will be essential for determining whether OmpA serves primarily as a structural scaffold for virulence or as a dynamic regulator of host–pathogen interactions.

### 2.3. Outer Membrane Vesicles: Distributed Effectors of Virulence and Interbacterial Communication

OMVs are nanosized lipid bilayer structures released from the surface of Gram-negative bacteria, including *A. baumannii*, that can function as mobile carriers of virulence determinants. Typically ranging from 20 to 300 nm in diameter, OMVs are composed primarily of outer membrane and periplasmic elements, including proteins, phospholipids and lipooligosaccharides, as well as select inner membrane and cytosolic proteins [8,47,59,60,61,62,63,64,65]. Rather than being passive byproducts of membrane turnover, OMVs are increasingly recognized as active secretion systems that extend the functional reach of a bacterium beyond direct cell–host contact. While the precise mechanisms for OMV formation remain incompletely defined, current evidence suggests that vesicle formation is driven by localized disruptions in peptidoglycan-outer membrane linkages and envelope stress. Accumulation of misfolded periplasmic proteins, turgor pressure and membrane curvature–inducing factors can promote membrane blebbing and vesicle release [66,67,68,69,70]. Turgor stress, secondary to the accumulation of misfolded periplasmic proteins, and peptidoglycan fragmentation has specifically been shown to promote membrane blebbing and vesicle release [71,72,73]. These observations suggest that OMV production is not random but instead may represent a regulated response to environmental and physiological stress, enabling bacteria to offload toxic materials while simultaneously exporting virulence factors.

Proteomic analyses of OMVs derived from *A. baumannii* revealed enrichment of multiple virulence-associated proteins, including those involved in host cell damage, immune modulation and iron acquisition [8,64,66,74,75,76]. Notably, OmpA is a prominent OMV-associated factor that retains its cytotoxic activity when delivered to host cells via vesicles [8]. The OMV mode of virulence factor delivery allows *A. baumannii* to decouple toxin delivery from direct bacterial contact, potentially enabling damage to distal host cells and contributing to tissue-level pathology. Emerging evidence further suggests that OMV composition is not static but selectively regulated. Differences in OMV protein content between wild-type and OmpA-deficient strains indicate that OmpA may influence vesicle biogenesis or cargo selection, possibly through interactions with peptidoglycan components such as diaminopimelate (DAP) [47,77]. This raises the possibility that OMVs are selectively packaged delivery systems shaped by key structural proteins.

Functionally, *A. baumannii* OMVs act as potent modulators of host immune responses. Exposure of epithelial and macrophage cell lines to OMVs induces strong, dose-dependent expression of pro-inflammatory cytokine genes including IL-1β, IL-6, IL-8, MIP-1α, MCP-1, and NLRP3 [76,78]. A reduced inflammatory response was observed with OMVs derived from OmpA-deficient strains, which further supports the idea that OMVs integrate multiple virulence signals into a coordinated response to the host. In vivo, OMVs contribute to lung pathology by promoting neutrophil recruitment and sustained inflammation. Transmission electron microscopy of lung tissue from infected mice revealed OMV budding from the bacterial surface, as well as hemorrhaging, necrosis and infiltration of polymorphonuclear immune cells in lung tissue [8]. OMVs inside lung tissue in infected mice regulated pulmonary consolidation for up to 48 h after initial infection via dose-dependent recruitment of neutrophils, as observed in bronchoalveolar lavage fluid [79,80]. Together, these findings position OMVs as key drivers of inflammation-mediated pathology, rather than passive byproducts of infection.

Beyond host interaction, OMVs play a critical role in interbacterial communication and the spread of antimicrobial resistance. OMVs can carry multiple classes of resistance determinants, including carbapenem-hydrolyzing β-lactamases (OXA-type enzymes), genetic material such as plasmids and small RNAs, and other factors that facilitate horizontal gene transfer. Specifically, *A. baumannii* OMVs have been documented to carry OXA-24 and OXA-58 carbapenemases and the blaNDM-1 metallo-β-lactamase gene, enabling susceptible bacteria to acquire resistance [40,81,82]. The ability of *A. baumannii* OMVs to carry these components from one bacterium to another contributes to the spread of carbapenem resistance, as well as this pathogen’s ability to survive and disseminate within the host’s systemic circulation [24,81,82,83,84]. Furthermore, this capacity enables *A. baumannii* to influence neighboring microbial populations, contributing to community-level resistance and adaptation within polymicrobial environments. In this context, OMVs function not only as virulence effectors, but also as vehicles for ecological competition and cooperation. Taken together, these observations support a model in which *A. baumannii* OMVs act as distributed virulence platforms, simultaneously contributing to host damage, immune modulation and interbacterial interactions. However, key questions remain regarding how OMV production and cargo selection are regulated during infection, and whether distinct OMV subpopulations serve specialized functions.

Resolving these issues will be essential for determining whether OMVs can be effectively targeted as part of anti-virulence strategies against *A. baumannii*. When used as a vaccine in mice, OMVs produce a strong antibody response, and protect against both pneumonia and sepsis when challenged with ATCC 19606^T^ as well as distinct clinical isolates [85,86]. Because OMVs naturally contain lipooligosaccharides (LOS), purified preparations carried high endotoxin activity (1.3 ± 0.01 × 10^5^ EU/μg), raising a safety concern in this approach when considering potential clinical application [85]. OMVs isolated from a *lpxD* mutant lacking LOS remained immunologically reactive and protected mice in a sepsis model while carrying significantly lower (6.3 × 10^−3^ ± 8.2 × 10^−4^ EU/μg) endotoxin levels, indicating the potential utilization of mutants when designing these vaccines [87]. Targeting OMVs is nonetheless complicated when considering the potential effects from the large number of virulent factors carried within them, and the signals governing selection of those factors remain undescribed [8,20,40,47].

### 2.4. Omp33-36: A Constrained Porin Linking Stress Tolerance, Intracellular Survival and Host Cell Death

Outer membrane protein 33-36 (Omp33-36) represents a functionally specialized outer membrane protein that integrates barrier function with virulence in *A. baumannii*. Omp33-36 appears optimized for restricted solute permeability and host interaction, contributing to adhesion, invasion, cytotoxicity and resistance to both antibiotics and environmental stressors [88,89]. Structurally, Omp33-36 is composed of 14 antiparallel *β*-strands forming a *β*-barrel, with an atypical periplasmic loop that partially occludes the porin channel [90,91]. This structural occlusion likely underlies the reduced permeability of the pore and suggests that Omp33-36 functions less as a transport conduit and more as a protective and regulatory interface with the environment.

A defining feature of Omp33-36 is its ability to directly induce host cell damage while simultaneously promoting *A. baumannii* intracellular persistence within host autophagosomes. Delivered to host cells via OMVs, Omp33-36 triggers destabilization of the mitochondrial electrochemical gradient triggering release of cytochrome c and subsequent activation of caspases 9 and 3, which lead to apoptosis [20]. However, this apoptotic signaling is coupled with disruption of autophagy: Omp33-36 induces autophagosome formation but blocks fusion of these autophagosomes with lysosomes, resulting in the accumulation of autophagosomes and associated markers such as p62 and LC3B-II. Due to the arrested autophagosomes being unable to proceed to lysosomal fusion and degradation, intracellular bacteria enclosed within them are sheltered from clearance. Apoptosis does not appear to directly enable this survival. Its role may lie in promoting eventual host cell death and tissue damage, which could facilitate dissemination once intracellular persistence has been established. The dual effect of inducing cell death while impairing cellular clearance mechanisms suggests that Omp33-36 enables *A. baumannii* to exploit host cell stress responses to enhance intracellular survival. Consistent with this model, deletion of *omp33-36* significantly reduces bacterial recovery from macrophages, indicating that the protein contributes to persistence within host cells rather than simply promoting cytotoxicity [20]. In addition to its role in intracellular survival, Omp33-36 contributes to broader infection dynamics including tissue invasion and systemic dissemination. Strains lacking *omp33* have considerably reduced adherence, invasiveness and cytotoxicity of lung epithelial and macrophage cell lines, as well as decreased virulence in a murine sepsis model [88]. These findings position Omp33-36 as a multifunctional determinant of host colonization and disease progression, likely acting in concert with other outer membrane proteins such as OmpA. Omp33-36 also plays a role in environmental resilience, particularly in resistance to disinfectants. Omp33-36 contributes to tolerance against benzalkonium chloride, a commonly used hospital disinfectant, suggesting that it supports survival both within the host and clinical environments [89]. This dual role highlights an important concept: virulence factors in *A. baumannii* often serve overlapping functions in both pathogenesis and environmental persistence, facilitating transmission and infection in healthcare settings.

The conservation of Omp33-36 across over 1600 strains with >98% sequence identity paired with strong immunogenicity supports its potential as a vaccine target [92]. The Omp34 variant of Omp33-36 was effectively recognized by IgM, IgA and IgG antibodies in patients infected with *A. baumannii* and elicits strong protective responses in murine models, including bactericidal activity and organ clearance [93,94]. The high conservation and immunogenicity of Omp33-36 further underscore this factor’s importance in virulence and indicate that it may be a promising candidate for the development of a broadly protective *A. baumannii* vaccine. However, as with other multifunctional virulence factors, it remains unclear whether targeting Omp33-36 alone would sufficiently disrupt infection or whether compensatory mechanisms would limit its effectiveness. Much of the activity of Omp33-36 occurs after delivery into host cells, so surface-directed antibodies would only engage a portion of the functional pool. The apoptotic and autophagosome maturation-arresting functions may be insufficiently affected [20]. The porin’s contributions to adhesion and invasion also overlap with those of OmpA, providing a potential compensatory route that a single-antigen immunization could not address [88]. Another concern is that reducing Omp33-36 expression is associated with imipenem resistance, and the porin was found to be under-expressed in 76% of a panel of 100 MDR and XDR *A. baumannii* clinical isolates [95,96]. Reported conservation also varies by analysis, with one study describing Omp33-36 as highly polymorphic and having strain-dependent transcription, making its application as a broad antigen difficult [97].

### 2.5. OmpW: A Context-Dependent Porin Linking Environmental Sensing, Iron Homeostasis and Virulence

Outer membrane protein W (OmpW) appears to function as a context-dependent interface between environmental sensing and virulence in *A. baumannii*. Structurally, OmpW forms an eight-stranded *β*-barrel with a predominantly hydrophobic channel, suggesting a role in the transport or binding of small hydrophobic molecules [98,99]. Interestingly, the periplasmic half of the channel is mostly hydrophilic, which may contribute to its ion channel activity, as shown in single channel conductance experiments [98]. This architecture distinguishes OmpW from more permissive porins and implies that it may act less as a general diffusion channel and more as a selective gateway that is responsive to environmental conditions.

A striking feature of OmpW is its highly dynamic and sometimes contradictory regulation under antibiotic stress. While some studies report decreased *ompW* expression in carbapenem- and colistin-resistant strains, the MDR isolate BAA-1605 exhibited a 3-fold increase in expression [100,101,102]. A recent study using in vitro colistin induction assays on clinical isolates obtained from a patient with a carbapenem-resistant *A. baumannii* infection demonstrates that *ompW* expression and OmpA production fluctuate during the acquisition and loss of colistin resistance. Transient *ompW* upregulation was observed with colistin exposure followed by loss of OmpW protein production in resistant populations and reappearance upon restoration of susceptibility [103]. *A. baumannii* colistin sensitivity may be partially due to the hydrophobic extracellular portion of the OmpW channel contributing to antibiotic binding, though one study noted unchanged colistin and imipenem minimum inhibitory concentration (MIC) values in a Δ*ompW* mutant [104]. Rather than representing experimental inconsistency, these findings suggest that OmpW participates in a dynamic adaptation strategy, where its expression is modulated to balance nutrient uptake and membrane permeability in the context of antibiotic exposure.

This trade-off between nutrient uptake and antibiotic resistance is particularly evident in the context of iron acquisition. Loss of OmpW, as was observed with colistin resistance, results in impaired iron uptake and disrupted iron homeostasis, indicating that OmpW is an important contributor to nutrient acquisition [103,104]. These observations highlight the potential of OmpW as an essential regulatory porin for iron uptake and homeostasis. The decreased production of OmpW under iron-limiting conditions, such as those encountered in the host, appears to be a paradox, with selective reduction of a protein required for nutrient acquisition at a time when the nutrient need is high. However, the reduction in OmpW in iron-chelated media may point to a adaptive response to the host environment that is often iron-limited and may possess antimicrobials which OmpW could potentially bind. Thus, the selective reduction in OmpW in the iron-limited host environment is likely an *A. baumannii* survival mechanism to minimize vulnerability to host defenses or antimicrobial agents [105]. This pattern highlights a broader principle in *A. baumannii* biology: virulence-associated functions are often governed by trade-offs between resource acquisition and survival under stress.

OmpW also contributes to host interaction and colonization in a context-dependent manner. Deletion of *ompW* reduces adhesion, invasion and biofilm formation without affecting bacterial growth, indicating that OmpW specifically supports host-associated phenotypes rather than general fitness [106]. Reduced virulence in animal models, including increased LD50 and decreased dissemination, further supports a role for OmpW in early-stage infection and tissue colonization [106,107]. Interestingly, OmpW production is reduced under hypoxic conditions, which are common in infected tissues, suggesting that its contribution to virulence may be temporally restricted to specific stages of infection. Together, these findings position OmpW as a regulatory porin that integrates environmental signals with virulence-related behaviors rather than a constitutive determinant of pathogenicity. The fluctuating expression of OmpW across variable antibiotic exposure, iron availability and oxygen levels suggests that OmpW enables *A. baumannii* to fine-tune its interaction with both the host and antimicrobial agents.

OmpW has also been assessed as a vaccine candidate. A study found the protein was more than 91% conserved across 14 PDR *A. baumannii* clinical isolates, and both passive and active immunization significantly protected mice in a sepsis model, reduced bacterial organ burden and suppressed inflammatory cytokines [86,108]. Additionally, the antisera demonstrated opsonophagocytic activity against distinct clinical isolates [86]. OmpW’s production, however, has been observed to fall in colistin-resistant strains, under iron limitation and under hypoxia, so the antigen may be least abundant under conditions encountered during infection [103,105,106]. Additionally, there is a possibility that suppressing the protein through a therapeutic could reduce colistin susceptibility, although the previously described Δ*ompW* mutant had unchanged colistin and imipenem MIC values [104]. The relationship between the porin and colistin activity must be better understood before this protein can be fully considered.

### 2.6. CarO: A Selective Porin at the Interface of Antibiotic Entry, Nutrient Sensing and Host Adaptation

Carbapenem-associated outer membrane protein (CarO) exemplifies how outer membrane proteins can simultaneously mediate antibiotic susceptibility, nutrient acquisition and virulence in *A. baumannii*. Initially identified through its role in carbapenem uptake, loss of CarO confers imipenem resistance, establishing it as a key entry pathway for this class of antibiotics [19]. Subsequent studies have however revealed that CarO is a selective porin whose substrate specificity creates an inherent trade-off between nutrient acquisition and antibiotic susceptibility. Two CarO subgroups (CarOa and CarOb) have demonstrated specific sensitivities towards imipenem based on their distinct structures [109]. CarO preferentially transports L-ornithine and related basic amino acids, and this specificity directly interferes with carbapenem permeability. Competition between imipenem and the physiological substrate L-ornithine for passage through CarO reduces antibiotic influx in variants such as CarOa, which transports ornithine; CarOb, by contrast, instead binds imipenem itself with roughly twice the affinity of CarOa [21,109]. This relationship highlights an important principle: antibiotic entry can be governed by evolutionary constraints imposed by nutrient transport, linking metabolic function to resistance phenotypes.

Beyond its role in carbapenem permeability, CarO contributes to infection through actions related to adhesion, invasion and biofilm formation. Deletion of CarO reduces adherence to and invasion of A549 human lung epithelial cells in vitro, indicating that it supports early stages of host colonization and tissue invasion [9]. In a murine peritoneal sepsis model, mutants lacking CarO required a higher infectious dose to cause lethality and showed reduced dissemination to internal organs, indicating impaired mechanisms of systemic infection. CarO upregulation during biofilm formation and under iron-limiting conditions further suggests that it is integrated into adaptive responses that promote persistence in both environmental and host-associated niches [105,110,111].

A notable feature of *carO* is its dynamic regulation in response to environmental stress. Culture of *A. baumannii* in high sodium chloride concentrations leads to osmotic stress and decreased expression of *carO.* This stress additionally leads to the upregulation of efflux transporters, which causes widespread release of CarO into the supernatant [112]. In contrast, mutants lacking the RNA chaperone Hfq show increased *carO* expression, likely reflecting an impaired ability to properly adapt to environmental changes leading to dysregulated stress responses [113]. Furthermore, stress induced by exposure to cool temperatures (15 °C compared to 37 °C) reduces CarO production by 80% [21]. The existence of other potential CarO regulatory activities is demonstrated in IPM-resistant clinical isolates that possess *carO* but do not produce the CarO protein [114]. These observations suggest that *A. baumannii* can fine tune membrane permeability by controlling CarO protein abundance to balance the need for nutrient uptake against protection against harmful compounds, including antibiotics. These findings position CarO as a tightly regulated multifunctional channel linking environmental sensing with survival and virulence-associated behaviors.

This regulatory and functional flexibility supports the hypothesis that CarO may serve as a sensor to detect entry into the host, particularly through its interaction with amino acids such as ornithine and arginine. These metabolites vary in concentration during processes such as wound healing and tissue remodeling, providing potential cues that signal host entry [115,116]. Additionally, ornithine accumulates in human skin as a part of normal aging due to post-transcriptional modifications in proteins such as collagen. This modification is exacerbated under specific pathological conditions [21,117]. By linking metabolite substrate availability to membrane permeability and downstream phenotypes, CarO may enable *A. baumannii* to adaptively transition between environmental survival and host-associated virulence states, contributing to pathogen persistence in a rapidly changing environment. Thus, CarO illustrates how a single outer membrane protein can integrate transport, sensing and regulatory functions to shape bacterial behavior.

Evidence for CarO as a potential therapeutic target is currently limited. Surface topology and linear B-cell epitope prediction have shown residues of CarO as a vaccine candidate, and CarO has been used alongside OmpA and zinc uptake component D (ZnuD) in a construct predicted to be antigenic, stable and to engage TLR 2 and TLR4 in docking simulations [118,119]. Neither study included immunization or infection experiments, and no in vivo protection data has been currently reported for CarO in this regard. Other factors may complicate development of an effective therapeutic. Loss of CarO has been associated with imipenem resistance, meaning a CarO-targeting vaccine may be least effective against the carbapenem-resistance isolates making this organism a priority pathogen [4,19,114].

## 3. Outer Envelope Components and Secreted Virulence Factors

**Figure 2:**
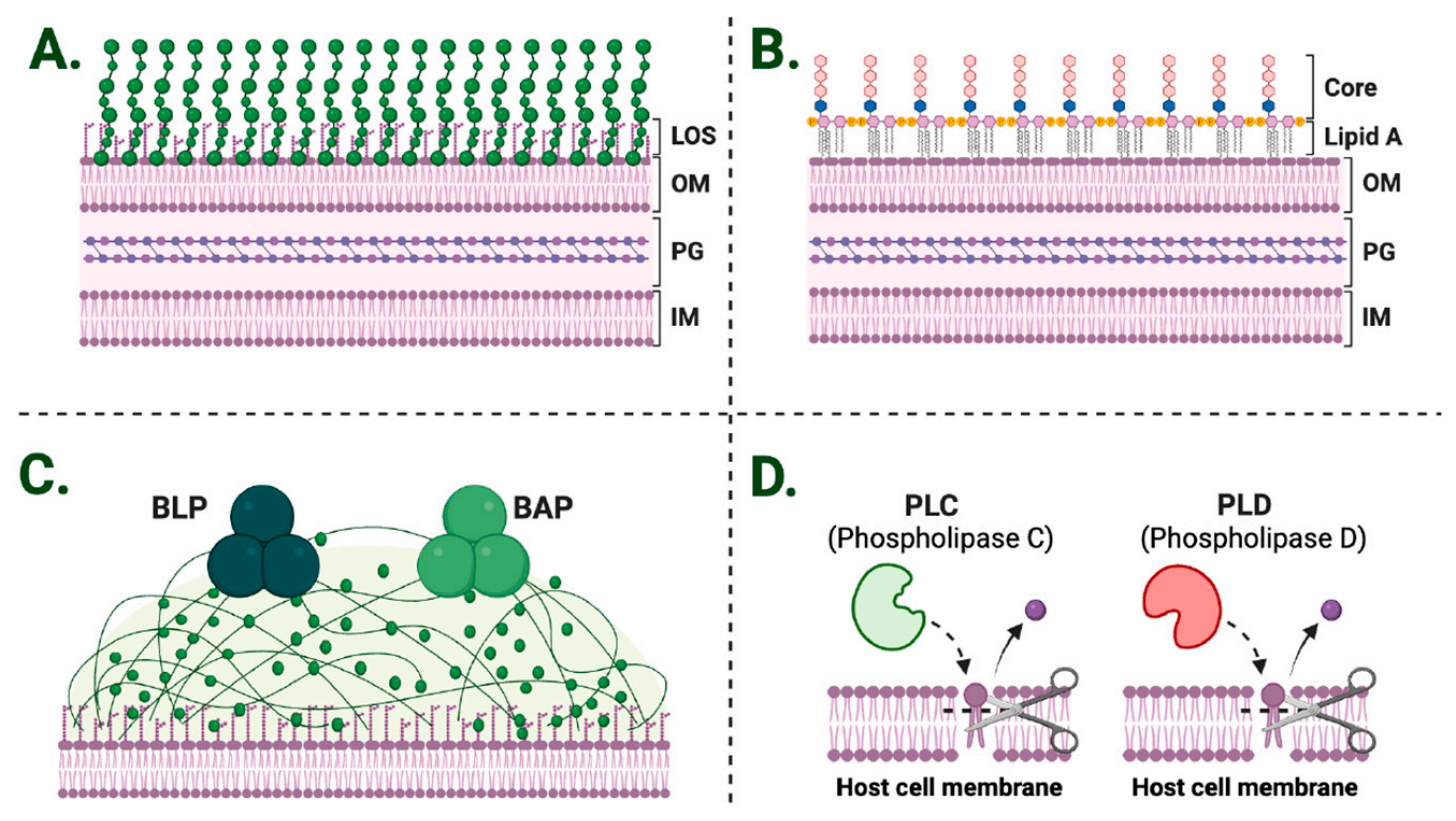
Schematic representation of major outer envelope components and secreted virulence factors of *A. baumannii*. (**A**) Lipooligosaccharide (LOS) is shown integrated into the outer membrane (OM), peptidoglycan (PG), and inner membrane (IM) layers of the bacterial cell envelope. (**B**) The detailed molecular architecture of LOS. (**C**) Biofilm-associated protein (Bap) forms a structural scaffold on the outer membrane to support biofilm development. (**D**) Phospholipase C (PLC) and phospholipase D (PLD) actively cleave phospholipids within the host cell membrane. Created in BioRender. Brancato, P. (2026) https://BioRender.com/llf61jw (accessed on 20 September 2026).

*A. baumannii* outer envelope components and secreted factors collectively form a critical network that sits at the interface between *A. baumannii* and its host environment, enabling both bacterial protection from host defenses and active manipulation of host tissues. These bacterial virulence factors include surface-exposed structures such as lipooligosaccharide (LOS) and capsule, as well as secreted or surface-associated proteins including phospholipases (PLC and PLD) and biofilm-associated proteins (BAP and BLP) (Figure 2). Rather than acting independently, these components contribute to a coordinated set of functions, including promotion of immune evasion, enhancement of adherence and biofilm formation, facilitation of tissue invasion and support for persistence during infection. Together, they illustrate how *A. baumannii* leverages its outer envelope not only as a physical barrier, but as a dynamic platform for host interaction and pathogenicity [10,120,121,122,123,124,125,126,127,128,129].

### 3.1. Lipooligosaccharide: A Structural Shield and Immunological Trigger Governing Virulence

Lipooligosaccharide (LOS), often still referred to as LPS in the literature, though *A. baumannii* characteristically lacks the repeating O-antigen units that define classical lipopolysaccharide (LPS), is a defining component of the outer membrane of *A. baumannii* and functions as both a physical barrier and a potent mediator of host–pathogen interactions. Structurally, LOS is composed of lipid A, which anchors the molecule in the outer membrane, a core oligosaccharide, and the distal O-antigen (Figure 2A) [11]. The dense, amphipathic nature of LOS confers protection against penetration of harmful molecules such as bile salts, disinfectants and antibiotics [11,129,130,131]. LOS-deficient *A. baumannii* strains display increased sensitivity to neutrophil-secreted lysozyme and lactoferrin, *β*-lactam drugs and disinfectants such as ethanol and chlorhexidine when compared to LOS-positive strains [132,133]. In this sense, LOS serves as a first line of defense, reinforcing the outer membrane as a barrier that protects against harsh conditions.

LOS is not merely structural component of the outer envelope; it is also a significant antigen that contributes to immune recognition. Lipid A is a potent activator of TLR4, triggering inflammatory responses that can both restrict infection and contribute to host damage. A mutant producing a severely truncated LOS showed decreased survival in a rat soft tissue infection model and normal human serum, as well as deficient growth in iron-limiting conditions, when compared to wild-type [120]. Paradoxically, complete loss of LOS leads to colistin resistance as these antibiotics target lipid A membrane disruption [121,134]. While LOS-deficiency may confer a competitive advantage during colistin treatment, multiple studies have shown LOS deficiency leads to reductions in in vitro proliferation, surface motility, biofilm production, as well as growth and dissemination in vivo [121,122,132,133,135,136]. The inhibition of lipid A production using LpxC inhibitors, which block the enzyme catalyzing the first committed step of lipid A biosynthesis, reduces bacterial burden and inflammatory cytokine levels in vivo, while enhancing susceptibility to immune clearance. In one study, the complete survival of mice was attributed to the prevention of an LOS–toll-like receptor 4 (TLR4)-mediated cytokine storm which often leads to hyperinflammation in tissue resulting in lethal sepsis [121,137]. Interestingly, the LpxC inhibitor used in this study had no effect on bacterial growth in vitro, highlighting the potential usefulness of antibacterial agents that are ineffective in vitro but exhibit strong efficacy in animal models [121]. This suggests that disrupting virulence-associated structures can be therapeutically effective even in the absence of direct bactericidal activity, and more broadly, this represents a paradigm shift toward anti-virulence strategies that target host–pathogen interactions rather than bacterial viability alone.

### 3.2. Biofilm-Associated Protein: A Structural Scaffold Driving Surface Colonization and Community Architecture

Biofilm-associated protein (Bap) is a key structural organizer of surface colonization by *A. baumannii*, enabling the transition from planktonic growth to stable, surface-attached communities. Bap is a high-molecular-weight (854 kDa) protein present on the bacterial surface of several pathogenic Gram-positive and Gram-negative bacteria including *A. baumannii* [138,139,140] (Figure 2). Bap contains a conserved central core region consisting of 3 to 50 identical tandem repeats which fold into an immunoglobulin-like three-dimensional structure, which is characterized by 70 to 100 amino acids arranged into seven anti-parallel *β* strands. These strands form two *β*-sheets which are stacked closely together into a compact configuration [128,141]. While Bap is structurally complex, its functional role can be distilled into a central purpose: act as a molecular scaffold that promotes the cell-surface and cell–cell interactions necessary for biofilm development.

Functional studies consistently demonstrate that Bap is required for the formation of mature, three-dimensional biofilm structures. Bap-positive strains produce dense and thick biofilms covering a significantly higher portion of a surface compared to Bap-negative strains, which produces thin biofilms with reduced volumes across a range of abiotic surfaces, including glass and medically relevant materials such as polystyrene, polypropylene and titanium [127,140]. Further genetic evidence provided by the transposon disruption of Bap demonstrates significantly impaired biofilm formation, with mutant biofilms reaching less than half the thickness of the wild type at 6 h and showing a continued decline in biovolume over time. While scanning electron microscopy of wild-type biofilms grown on polystyrene and titanium reveals the formation of three-dimensional towers with water channels, the Bap mutant forms disorganized monolayers that lack mature structures [127]. These findings suggest that Bap provides the physical framework necessary for the emergence of complex biofilm architecture. Inhibition of Bap through antibody interference similarly disrupts early biofilm formation, indicating that Bap is not only a structurally important but is also functionally required during initial attachment and subsequent biofilm maturation [142]. Notably, Bap appears to preferentially mediate adhesion rather than invasion. Strains expressing Bap show enhanced binding to human bronchial epithelial and neonatal keratinocyte cells compared to the mutant but do not display increased invasion [127]. This specialization in surface colonization rather that host–cell invasion highlights an important division of labor among virulence factors, where some promote entry into host cells while others, such as Bap, support extracellular persistence and host niche establishment.

An additional, and somewhat counterintuitive, feature of Bap is its influence on cell surface hydrophobicity. Despite predictions of hydrophilicity, Bap expression markedly increases the hydrophobicity of the bacterial cell surface as a whole as demonstrated by enhanced hydrocarbon partitioning [127,143]. This may be attributed to the hydrophobic nature of the Ig-like *β*-sandwich structure that Bap is predicted to adopt. This property likely facilitates attachment of bacteria to abiotic surfaces, including plastics and medical devices, reinforcing the idea that Bap contributes to environmental persistence in clinical settings.

Genomic analyses of *A. baumannii* strains demonstrate that Bap is part of a broader family of related proteins that contribute to biofilm formation and host cell adherence [128]. Bap has several structural variants, defined by differences in the Ig-like domains and C-terminal regions, as well as two Bap-like proteins (BLP1 and BLP2). *A. baumannii* AYE deletion mutants deficient in these proteins (Δ*blp1* and Δ*blp2*) have impaired biofilm formation as well as reduced adherence to A549 pulmonary epithelial cells [128]. Taken together, Bap and BLPs emerge as central architectural determinants of biofilm formation, linking surface adhesion, structural organization and persistence. Given the conserved functional role and surface accessibility of Bap, this molecule represents a promising target for strategies aimed at disrupting biofilms and limiting chronic infection. Antigenic variation, however, could pose as a limiting factor in development of a therapy. Due to Bap’s large size and varying number of tandem repeat regions between strains, an antibody developed only recognizes a defined epitope [128,140]. The monoclonal antibody 6E3 recognized an epitope conserved in 41% of *A. baumannii* isolates from a military outbreak, and subsequent work by the same research group evaluating a larger bank of clinical isolates found similar reactivity [127,140]. These figures describe the prevalence of a single epitope rather than of the protein itself, meaning antibody-based intervention may fail against isolates not expressing the target epitope. Additionally, *A. baumannii* possesses highly redundant mechanisms for biofilm formation and adherence including Csu pili and OmpA, and has independent contributions from BLP1 and BLP2, leaving functional compensatory routes intact when using anti-virulence therapies [53,128,144].

### 3.3. Capsule: A Tunable Shield Governing Immune Evasion, Fitness and Virulence Trade-Offs

The capsular polysaccharide (CPS) of *A. baumannii* functions as a primary interface site between the bacterium and the host immune system, enabling both protection from host defenses and modulation of virulence. CPS is composed of high-molecular-weight oligosaccharide repeats (K-units), which contain varying compositions of 2–8 linear or branched sugars. Biosynthesis of these structures is encoded by genes within the K locus (KL), which exhibit extensive genetic diversity [145]. Structural analyses reveal substantial heterogeneity among capsule types. The K2 capsule (KL2) contains common sugars along with a unique pseudaminic acid not found in other types of capsules, whereas the K4 capsule is composed entirely of amino sugars [146,147]. Other types, such as K35 and K15, include amino and diamino uronic acids [145]. These diverse structures translate into clinically relevant phenotypes, with certain capsule types (particularly KL2) having a strong association with multidrug resistance, increased disease severity, and poor clinical outcomes, including higher intensive care unit admission rates and increased mortality [148,149]. While only around 66 unique K-unit structures are predicted by bioinformatic analyses, there are over 237 KL types described among *A. baumannii* strains, suggesting that *A. baumannii* employs a combinatorial strategy of genetic variation and structural convergence to maximize capsular diversity in order to optimize immune evasion and bacterial persistence [145,150].

At the genetic level, capsule diversity is underpinned by a highly organized and tightly regulated biosynthetic system. The K locus has a modular architecture, with a conserved 5′ region encoding export and assembly proteins such as Wza, Wzb, Wzc as well as a conserved 3′ region encoding for UDP-sugar synthesis. A variable central region encodes glycosyltransferases as well as the Wzy polymerase required to build and assemble capsule repeating units [151,152,153]. Capsule production is further regulated by the BfmRS two-component transcriptional regulatory system, which links environmental cues, including antibiotic exposure, to increased capsule synthesis [151,154]. This BfmRS-driven upregulation of capsule enhances serum resistance, antibiotic tolerance, and virulence, whereas its disruption leads to reduced fitness and increased susceptibility [151,155]. In combination, these observations suggest that capsule diversity and expression are not static features, but part of a regulated adaptive strategy that directly shapes clinical outcomes.

Functional studies consistently demonstrate that CPS is essential for immune evasion and in vivo survival. Disruption of capsule-associated genes broadly reduces bacterial fitness, increases immune-mediated clearance, and attenuates virulence. Genome-wide screens identify capsule-associated genes as critical for persistence, with disruptions resulting in substantial fitness defects in vivo [156]. Mutation of key biosynthetic genes, such as *wzc* or *wza*, leads to capsule loss, increased susceptibility to complement-mediated killing, enhanced phagocytosis and dramatically reduced virulence, with up to an 8-log decrease in bacterial burden in lung infection models [13,157,158,159]. Alternately, subtle structural modifications, such as deletion of a single branch sugar via *gtr6* disruption, can enhance immune evasion and increase virulence, demonstrating that capsule function is highly sensitive to fine structural changes [160]. Additionally, specific capsule types can differentially inhibit complement membrane attack complex formation while other capsule types remain susceptible, indicating functional specialization among capsule variants [12]. These data highlight the important role that a finely tuned capsule structure plays in *A. baumannii* infections in vivo.

Capsule abundance further modulates virulence in a dose-dependent manner. Hypercapsulated (mucoid) strains exhibit reduced C3b deposition, decreased phagocytosis and uptake by both mouse macrophages and A549 cells, respectively, and increased lethality in *G. mellonella*. Whereas capsule-deficient or low-capsule variants have increased immune recognition and clearance [161]. The transition from highly capsulated opaque to low-capsulated translucent colonies is controlled by a LysR-type regulator, ABUW_1132, whose overexpression reduces virulence in a mouse model correlating with a decrease in total capsule quantity [162,163]. These studies suggest a strong relationship between capsule mass and composition with immune evasion and host virulence in *A. baumannii*.

Of note, capsule-mediated virulence is governed by trade-offs. Increased capsule production enhances immune evasion and antibiotic resistance but can reduce adhesion, biofilm formation and cellular uptake [164,165,166]. Conversely, capsule-deficient mutants exhibit improved surface attachment and transformation efficiency but reduced virulence. These opposing effects support a model in which CPS functions as a tunable virulence determinant, dynamically adjusted to balance environmental persistence with host survival. Adaptive studies further reinforce this concept. Prolonged interaction with host cells positively selects for hypermucoid variants with enhanced resistance but diminished adhesion and biofilm capacity, suggesting that capsule remodeling contributes to within-host evolution and niche adaptation [166]. Regulation of capsule phase variation, such as transitions between opaque (high capsule quantity) and translucent (low capsule quantity) phenotypes mediated by transcriptional regulators like ABUW_1132, further highlights the role of CPS in phenotypic switching and environmental adaptation [162,163].

From a therapeutic perspective, the surface exposure and unique composition of *A. baumannii* capsules make them potential targets for antimicrobial development. Antibody-mediated opsonization of *A. baumannii* leads to enhanced human neutrophil killing in vitro and increased bacterial clearance in a rat soft-tissue infection [167]. A phage-derived capsule depolymerase selectively degraded K45 and K30 CPS and reduced serum survival of *A. baumannii*. This phage was nontoxic to A549 cells and did not lead to emergence of resistant strains after overnight exposure, indicating safety and viable translational relevance [168]. Another phage-derived CPS depolymerase (B3gp42) selectively degraded K2 and protected *G. mellonella* larva from infection [169]. However, the extensive diversity of CPS among different *A. baumannii* clinical isolates limits the effectiveness of broadly applicable antimicrobial strategies, indicating that successful interventions will require either multi-target approaches or precision therapies guided by rapid diagnostics.

### 3.4. Phospholipases: Enzymatic Effectors Linking Membrane Disruption, Host Interaction and Virulence Redundancy

Phospholipases (PLs) function as direct enzymatic effectors of host cell damage in *A. baumannii* infections, enabling invasion through targeted disruption of host membrane integrity. By hydrolyzing available ester bonds within host phospholipids, these enzymes destabilize host cell membranes and generate bioactive lipid products that modulate apoptosis, membrane trafficking and immune signaling [170,171,172]. Different classes of PLs hydrolyze distinct bonds in phospholipids and are thus classified into three major groups, termed A, C and D phospholipases. Phospholipase As (PLAs) are lysophospholipases that cleave off an acyl chain from the glycerol backbone at the sn-1 (PLA1) or sn-2 (PLA2) positions. However, the role of PLA in *A. baumannii* survival and infection remains unclear. Alternately, the phosphodiesterases phospholipase C (PLC) and phospholipase D (PLD) target the glycerophosphate bond and polar head group, respectively, and are key contributors to *A. baumannii* virulence [123,125] (Figure 2).

PLCs in *A. baumannii* drive epithelial cell invasion, cytotoxicity and virulence in vivo [10,123,129]. *A. baumannii* ATCC 19606^T^ produces two iron-regulated phosphatidylcholine-specific PLCs (PC-PLCs) encoded by *plc1* and *plc2*, which display hemolytic activity against sheep, human and horse erythrocytes [10]. The hemolytic activity of these enzymes positively correlates with membrane phosphatidylcholine content, directly linking substrate availability to cytotoxic potential. Importantly, functional studies indicate that PLC1 and PLC2 operate in a partially redundant but collectively essential manner. Double mutants lacking both enzymes show markedly reduced hemolytic activity and virulence, whereas the PLC2 single mutant retains near wild-type pathogenicity in *G. mellonella* larvae [10]. This pattern suggests a broader principle: virulence-associated enzymatic systems in *A. baumannii* may be buffered by redundancy, ensuring that loss of a single factor does not abolish pathogenic capacity. Additionally, stress-induced upregulation of *plc1* under ethanol exposure indicates that PLC expression is responsive to environmental cues [123]. Thus, these data support a model in which the membrane-disrupting activity of *A. baumannii* is dynamically governed by the deployment of phospholipases in response to environmental conditions.

Similar to PLCs, PLD enzymes contribute to virulence but exhibit a more complex interplay of redundancy and specialization. *A. baumannii* encodes three PLDs (PLD1–3), which together support epithelial invasion and resistance to cationic antimicrobial peptides [124,125,173]. In *A. baumannii* ATCC 19606^T^ these three PLDs synergistically contribute to A549 epithelial cell invasion and mortality of *G. mellonella* larvae [125]. Compared to wild-type, single (Δ*pld*1, Δ*pld*2, Δ*pld*3) and double (Δ*pld1*/Δ*pld2*, Δ*pld1*/Δ*pld3*, Δ*pld2*/Δ*pld3*) *pld* mutants showed minimal attenuation; however, the triple Δ*pld1*/Δ*pld*2/Δ*pld*3 knockout displayed significantly reduced virulence, establishing the construct of functional overlap within this enzyme family. Notably, a single *pld2* mutation led to a reduction in invasion that was unrelated to A549 cell adhesion [125]. Mutation of *pld2* in the clinical isolate 98-37-09 resulted in similar diminished invasion of BEAS-2B bronchial epithelial cells and HeLa cervical cancer cells, pointing to the unique importance of PLD2 in cell invasion despite being a close paralog to *pld1* with a shared domain architecture [124,125]. While there is some redundancy among some PLD functions, this distinction highlights the potential for a division of labor within phospholipases, where individual enzymes may contribute differently to discrete stages of infection.

Beyond host interaction, PLDs also influence bacterial membrane composition. In the ATCC 19606^T^ strain*,* PLD1 plays a role in cardiolipin (CL) synthesis and PLD2 and PLD3 are involved in monolysocardiolipin (MLCL) synthesis [173]. CL, MLCL and phosphatidylethanolamine (PE) are abundant membrane phospholipids in *A. baumannii* strains ATCC 19606^T^ and AYE, with phosphatidylglycerol (PG), acyl-PG and lyso-PE being present in smaller quantities [174]. Alterations in membrane lipid composition affect susceptibility to cationic antimicrobial peptides such as colistin and polymyxin B, suggesting that PLDs contribute to intrinsic resistance by modulating membrane properties. Indeed, triple PLD mutants show increased sensitivity to these antibiotics, linking bacterial phospholipid metabolism to antimicrobial resistance [173].

Together, PLC and PLD enzymes illustrate how *A. baumannii* employs phospholipases as a multifunctional virulence system that plays a pivotal role in host damage, environmental sensing and membrane adaptation. PL redundancy ensures robustness, while specialization allows for fine-tuned contributions to invasion, cytotoxicity and resistance. Importantly, phospholipases also represent promising therapeutic targets. Inhibition of PL activity using compounds such as miltefosine reduces host cell damage and tissue pathology in infection models, even in the absence of direct antibacterial activity [175,176]. This supports the emerging concept that targeting specific virulence mechanisms rather than global bacterial viability may be an effective strategy against multidrug-resistant pathogens. Two considerations should be taken into account when discussing an effective strategy. As previously seen, PL has redundancy, with effects appearing only when all three PLDs are addressed [125]. This may explain why miltefosine, a phosphatidylcholine analogue, demonstrates effects across all treated strains when loss of an individual enzyme does not [175,177]. Addressing redundancy could lead to unwanted host engagement. Phosphatidylcholine is abundant in eukaryotic membranes, and PLD3 is found in eukaryotes as well [10,125]. An agent that is narrow enough to not affect eukaryotic cells may therefore be unable to address PL redundancy. Whether this tension can be resolved is a central question for this target.

## 4. Pili and Fimbriae

**Figure 3:**
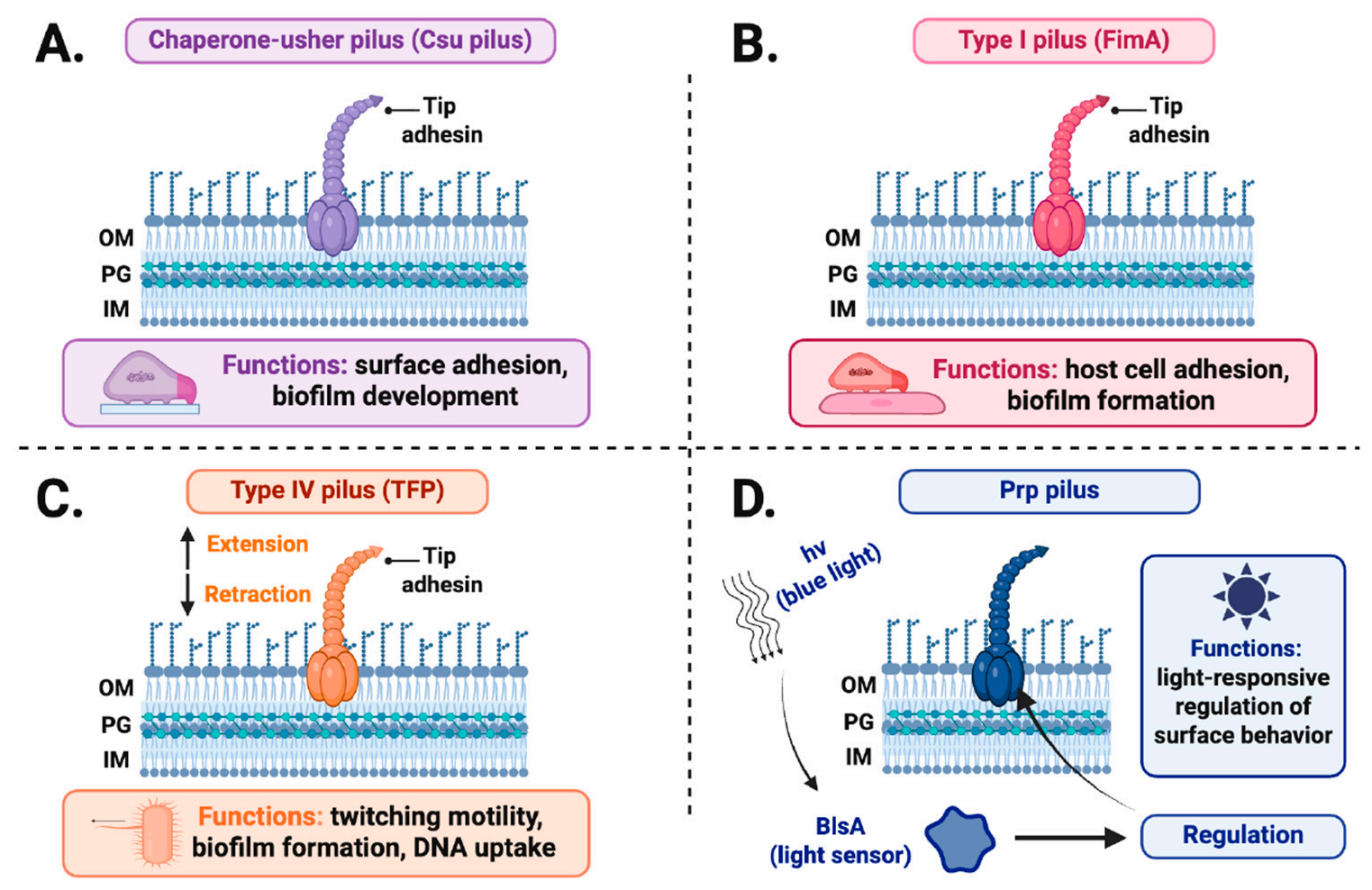
Schematic representation of major pili and fimbriae of *A. baumannii* linked to virulence. (**A**) The Csu chaperone-usher pili assembly system mediates attachment to surfaces. (**B**) A major subunit of Type 1 pilus FimA assists in host tissue colonization and adherence (**C**) Type IV pili (T4P) extends and retracts to drive twitching motility and facilitate natural transformation. (**D**) The Prp pilus is regulated by blue light through the blue-light photoreceptor BlsA. Created in BioRender. Brancato, P. (2026) https://BioRender.com/rqq8whd (accessed on 20 September 2026).

Pili and fimbriae are hair-like, filamentous appendages located on the surface of many Gram-positive and Gram-negative bacteria that serve as key mediators of host and surface interactions. In Gram-negative organisms, major pilus systems, including chaperone–usher pili, type I pili, and type IV pili, play key roles in virulence (Figure 3). The structures of these pili are typically composed of non-covalently assembled pilin subunits that form extracellular polymers with specialized adhesive and mechanical properties [14,15,178]. *A. baumannii* encodes multiple pili systems, encoded by genes such as *pilA*, *prpA* and *csuA*/*B* whose products contribute to virulence by promoting adhesion to biotic and abiotic surfaces, biofilm formation, twitching and surface motility, and horizontal gene transfer [144,179,180,181]. Notably, pili-mediated adhesion often acts in concert with biofilm-associated proteins such as Bap, where the initial surface attachment by pili facilitates the subsequent development of mature, structured biofilms [140,181].

### 4.1. Chaperone–Usher Pili: Specialized Adhesion Machinery for Surface Colonization and Biofilm Initiation

Chaperone–usher pathway (CUP) pili represent a specialized adhesion system that enables *A. baumannii* to efficiently colonize abiotic surfaces and initiate biofilm formation. Bacterial CUP systems are broadly categorized into alternative, classical and archaic groups, which are further categorized into six phylogenetic clades (α, β, γ, κ, π, and σ) based on usher protein sequence [182]. Csu pili of *A. baumannii* belong to the archaic CUP family and are encoded by a six-gene operon (*csuA*/*B*, *csuA*, *csuB*, *csuC*, *csuD*, *csuE*) that is responsible for the production of the major subunit of the pilus (CsuA/B), the chaperone (CsuC) and usher (CsuD) proteins, and the tip adhesin (CsuE) protein which work together to coordinate pilus assembly and surface presentation [181]. Assembly of the Csu pilus proceeds through a highly coordinated chaperone–usher mechanism in which pilin subunits (composed of a CsuA/B) are stabilized in the periplasm by the chaperone CsuC and subsequently delivered to the outer membrane usher CsuD for polymerization and eventual secretion through a process that has yet to be fully characterized [154,183,184]. Evidence indicates that donor strand complementation (DSC) drives self-assembly of pilin subunits into the growing fiber by insertion of the N-terminus of one pilin subunit into the non-polar pocket of an adjacent unit, forming a stable extracellular polymer. This suggests that the system possesses a degree of intrinsic robustness, allowing pilus formation to proceed efficiently regardless of external environmental conditions.

The Csu pilus is specifically adapted for adhesion to abiotic surfaces, a property that is central to *A. baumannii* persistence in clinical environments. This specialization is largely mediated by the tip adhesin CsuE, which directly interacts with environmental surfaces and determines binding specificity. Notably, CsuE features a hydrophobic binding domain formed by three finger-like loops, rather than the two-loop arrangement typical of related adhesins [185]. This additional hydrophobic projection enhances binding to hydrophobic materials while reducing adhesion strength to hydrophilic surfaces such as glass. Mutations in this unique third finger leads to reduced biofilm formation, while mutations in the conserved fingers nearly abolish it [185]. The unique three-loop domain likely increases the strength of *A. baumannii* adhesion to hydrophobic surfaces, such as medical devices, enabling biofilm formation that enhances the pathogen’s tolerance to environmental stress and persistence in the hospital setting [185,186]. Beyond its structural contributions, CsuA/B has also emerged as a promising vaccine candidate. CsuA/B exhibits high sequence conservation, with a >99% identity and ≥98% query coverage across nearly 3000 *A. baumannii* strains [187]. Mice immunized with CsuA/B alone achieved a 37.5% survival against the minimal lethal dose of *A. baumannii* ATCC 19606^T^, suggesting it may be most effective as part of a multicomponent vaccine strategy [187]. Additionally, a pan-genome analysis placed the *csu* pilus assembly cluster in the group of clusters prone to frequent gain and loss between isolates, with a relatively higher incidence of *csu*-deleted isolates among respiratory samples within clonal complex 2 [188,189]. This means a vaccine may be unable to cover a meaningful fraction of isolates, which could explain its need for a multicomponent strategy. Another issue is the loss of *csu* in isolates not leading to loss of function, with there being reported retention of short surface pili and enhanced attachment to bronchial epithelial cells [188,189]. This may explain the increased number of *csu*-deficient strains at respiratory sites, meaning therapies targeting *csu* may be most valuable in preventing device colonization, rather than an established respiratory infection.

### 4.2. FimA and Type I Pili: Mechanical Adhesion Systems Linking Surface Attachment to Immune Targeting

Type I pili in *A. baumannii* are mechanically specialized adhesion systems that support stable bacterial attachment within the host and on abiotic surfaces under dynamic conditions. Similar to the well-characterized pili found in *E. coli*, which comprise approximately 1000 subunits and a FimH tip, the *A. baumannii* homolog has an ability to transform from a coiled structure to an extended fiber under force, increasing the bacterium’s resistance to shear stress in environments like the respiratory tract and bloodstream [190,191,192]. In this context, FimA is not simply a structural subunit but a key determinant of mechanical resilience, enabling *A. baumannii* to sustain adhesion under disease-relevant conditions. Consistent with this role, *fimA* is upregulated during pellicle and biofilm formation, linking type I pili to surface-associated growth and persistence [193,194].

Beyond its structural role, FimA has emerged as a highly immunogenic and conserved antigen making it a favorable candidate for vaccine development. A reverse vaccinology approach found FimA (ABAYE2132) to be a prime vaccine candidate due to its > 99% sequence conservation across multiple *A. baumannii* strains [195]. Functional studies further support this, demonstrating that passive immunization with anti-FimA antibodies confers strong protection in murine models, including 100% survival and significant reductions in bacterial burden across multiple organs [196]. Another murine sepsis model that employed FimA as a subunit vaccine showed a 50% survival after challenge with a lethal dose of *A. baumannii*. Interestingly, 62% of mice were protected when immunized with both FimA and CsuA/B [187]. A separate model involving the active immunization of mice with a quadrivalent cocktail consisting of FimA, CsuA/B, BauA and HemTR antigens showed even greater survival rates (87.5%) when challenged with the ABI022 and ABI038 strains, which are known to be highly virulent with increased biofilm formation and cell adhesion, respectively [193,197]. Another in vivo model demonstrated 100% protection against *A. baumannii* infections in mice passively immunized with anti-FimA IgY antibodies [198]. Notably, antibody treatment targeting FimA disrupts adhesion, reducing bacterial attachment to epithelial cells by approximately 40% [196]. This reinforces the idea that FimA contributes directly to host colonization. However, variability in protection across studies suggests that FimA alone does not fully account for attachment-associated virulence, but rather that FimA acts as part of a multicomponent adhesion network. Variability in genetic makeup among strains must also be addressed. Using the same quadrivalent antisera, passive immunization protected 83.3% of mice infected with the *A. baumannii* ABI038 strain but only 16.66% of those infected with ABI022, a strain missing the *hemTR* gene entirely [197]. Protection therefore depends on whether the isolate carries the targeted antigens, similar to the situation previously described for Csu. Antigen selection for pili-directed vaccines must carefully account for genetic content of prevalent lineages, and once again underscores the importance of rapid diagnostic testing during infection.

The current thinking is that FimA functions as both a mechanical stabilizer of adhesion and a prominent immunological target. The roles of the FimA pilus in adhesion, pellicle formation, biofilm formation and resistance to shear stress, all of which enhance adherence to medical devices, demonstrate an association of this pilus with virulence. The exact mechanism through which FimA specifically contributes to biofilm and pellicle formation, however, has not been described and the disparity in FimA’s efficacy as a vaccine candidate in different studies indicates the need for further research.

### 4.3. Type IV Pili: Dynamic Motility Systems Linking Surface Exploration to Virulence

Type IV pili (TFP) are dynamic appendages that enable *A. baumannii* to actively explore and adapt to its environment, which distinguishes them from the more structurally stable CUP. TFP play a major role in twitching motility via cycles of extension, attachment and retraction driven by coordinated assembly and disassembly of pilin subunits [199,200]. This motility is powered by a conserved machinery in which PilA forms the major pilin subunit, PilD processes pilin precursors and PilT mediates retraction [201]. In addition to its role in motility, PilA has been associated with biofilm formation, adhesion and natural competence in *Acinetobacter*, suggesting that TFP contribute to multiple processes relevant to pathogenesis [180,202]. The specific role of TFP in *A. baumannii* infection is not clearly defined. While motility and biofilm formation are often linked to virulence in other bacteria, their contribution in *A. baumannii* appears to be context-dependent and incompletely understood [203,204].

PilA is strongly associated with motility in *A. baumannii* isolates, highlighting its role as a key determinant of (TFP)-mediated movement. In one study of 87 clinical and non-clinical isolates, all 11 non-motile strains lacked *pilA.* In contrast, all hypermotile isolates possessed the gene, indicating a strong correlation between *pilA* and motility capacity [203]. Building on this, emerging evidence suggests that TFP-mediated motility enhances virulence by promoting surface colonization and host interaction. Hypermotile derivatives of *A. baumannii* exhibit increased adhesion to human alveolar epithelial cells and greater virulence in a *Caenorhabditis elegans* infection model [205,206]. Together, these findings support a model in which motility facilitates efficient niche exploration and colonization. However, because motility can still occur in the absence of *pilA*, additional mechanisms likely contribute, underscoring the complexity of motility regulation and its relationship to virulence in *A. baumannii*.

### 4.4. Light Sensing: Environmental Integration of Surface-Associated Functions

*A. baumannii* has evolved to detect and react to extracellular cues as part of its ability to persist inside the host and in the environment. While light is classically associated with photosynthetic organisms, it is increasingly recognized as a regulatory signal influencing the physiology of non-photosynthetic bacteria with impacts on a number of processes such as motility, biofilm formation, and stress responses [207,208,209]. These light-modulated responses are mediated by conserved photoreceptor domains, including blue-light sensing using flavin (BLUF), light-oxygen-voltage and photoactive yellow protein, which enable bacteria to detect and respond to changes in light conditions [207,209].

In *A. baumannii*, light sensing is mediated by the BLUF-domain photoreceptor BlsA, which binds flavin adenine dinucleotide (FAD) as a light-absorbing cofactor [207,210]. Production and activation of BlsA under specific environmental conditions, particularly at lower temperatures (24 °C), represses downstream expression of the photo-regulated pilus (*prpABCD*) operon, linking light perception directly to surface-associated behaviors [179]. Disruption of *prpA*, which encodes a major type I pilin subunit of one of the four *A. baumannii* type I gene clusters, also results in reduced virulence in the *G. mellonella* infection model, indicating that BlsA-dependent light-responsive pilus expression contributes functionally to pathogenicity [211]. Genomic analyses further suggest that light-sensing systems may be conserved across *Acinetobacter* species, as homologs of the *prp* operon have been identified in multiple strains [179].

Notably, the expression of *A. baumannii* light-regulated genes is highly context-dependent, with temperature acting as a critical modulator. Transcriptional expression of *blsA* is drastically reduced, and BlsA sensing activity is abolished at 37 °C, thereby diminishing blue light-dependent regulation of virulence-associated functions under these human host temperature conditions [179,210,212]. This temperature-dependent behavior suggests that light sensing may be most relevant in environmental or transitional niches rather than the temperature imposed by the host. This possibility is supported by the observation that *prpA* mutation does not affect light-regulated motility or biofilm functions when bacteria are cultured at 37 °C [179]. This observation indicates that these two responses are mediated by cellular mechanisms and light regulatory functions that operate under host temperature conditions that remain to be identified and characterized.

The role of the light stimulon active at 37 °C was further extended by the observation that light also regulates, by an unknown mechanism, the expression of conjugative functions coded for by the *A. baumannii* ATCC 17978 pAB3 plasmid [213]. Incubation at 37 °C under dark conditions enhanced plasmid conjugation, while an isogenic plasmid-cured derivative displayed significantly enhanced surface motility. These observations indicate that pAB3 and the evolutionarily related plasmid pAB5, present in the *A. baumannii* UPAB1 isolated from a urinary tract infection, serve not only to transfer resistance genes and global regulators but also to confer surface motility [213]. Whether surface motility depends on plasmid-mediated regulators or the formation of surface-exposed conjugative pili, which play a role in biofilm biogenesis, remains to be explored.

The integration of light and temperature signals through light sensors exemplifies the broader principle that *A. baumannii* employs multi-signal regulatory systems to fine-tune virulence-associated behaviors across diverse environments. Rather than acting as a simple light-dependent on/off switch, the BlsA-Prp system appears to modulate interactions at the bacterial surface in response to environmental context, potentially facilitating transitions between environmental reservoirs and host-associated conditions, while additional uncharacterized light-regulated virulence-associated functions operate under host conditions. The virulence factors described in Section 2, Section 3 and Section 4 are summarized in Table 1.

## 5. Coordinated Virulence

**Figure 4:**
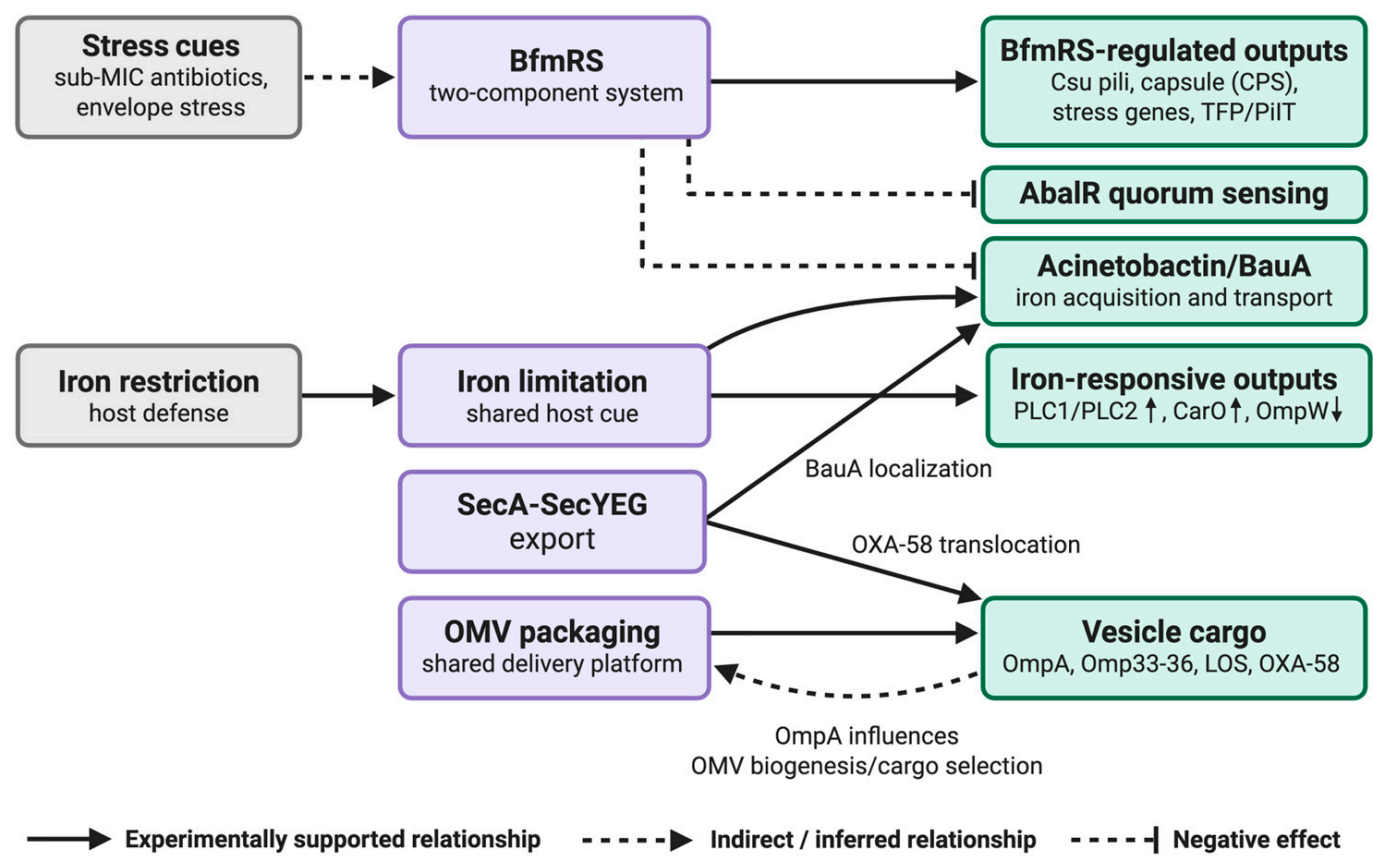
Schematic representation of major regulatory and functional connections of *A. baumannii* virulence factors. This scheme illustrates the shared regulation that BfmRS, iron limitation, outer membrane vesicles (OMV), and SecA translocase protein export pathway have on chaperone-usher (Csu) pili, capsular polysaccharide (CPS), stress genes, type IV pili (TFP/pilT), AbalR quorum sensing system, the acinetobactin/BauA iron acquisition pathway, phospholipase C (PLC1/PLC2), carbapenem-associated outer membrane protein (CarO), outer membrane protein W, A, and 33-26 (OmpW, OmpA, Omp33-36), as well as lipooligosaccharide (LOS) and oxacillinase-58 (OXA-58) after responding to environmental stress cues. Created in BioRender. Brancato, P. (2026) https://BioRender.com/ubsymsi (accessed on 20 September 2026).

Experimental evidence indicates that *A. baumannii* virulence factors can be linked to each other in two major ways. Some are co-regulated and respond to a shared transcriptional or environmental input, while others are physically localized together and share an export mechanism. Additionally, they may be packaged into the same vesicle despite not sharing a regulator [16,155]. The BfmRS two-component system, iron limitation as a cue, and OMVs as an export platform account for much of the coordination elucidated to date (Figure 4) [8,23,155].

### 5.1. BfmRS: A Node Linking Adhesion, Capsule and Resistance

BfmRS consists of the response regulator BfmR and the sensor kinase BfmS, which additionally acts as a phosphatase and restrains BfmR activity [214]. This system was first identified in *A. baumannii* ATCC 19606^T^ as being required for expression of the *csu* chaperone–usher operon and for biofilm formation on abiotic surfaces, placing the pilus described in Section 4.1 under the same regulatory input as biofilm development [154,181]. BfmR also drives capsule biosynthesis, and this output is amplified by sub-inhibitory exposure to antibiotics, coupling drug stress to increased CPS production, serum resistance and virulence, noted in Section 3.3 [151]. Broader analyses link BfmRS to envelope homeostasis, antibiotic resistance and virulence, with loss of this system resulting in defects to capsule production, serum survival and resistance phenotypes simultaneously [155]. Consistent with this, a Δ*bfmR* mutant demonstrated dysregulation of oxidative, osmotic and misfolded-protein stress responses along with reduced *csu* pilus and CPS gene expression [214]. There was also altered expression of acinetobactin synthesis and transport genes and altered type IV pilus production, with BfmR binding to several stress-related promoters [214]. BfmRS additionally affects quorum sensing, with a Δ*bfmR* mutant showing reduced biofilm and pellicle formation attributed to low *csu* expression while increasing *abaI* and *abaR* expression, indicating that BfmR normally suppresses the AbaIR quorum sensing system [215]. A Δ*bfmS* mutant in the same study had reduced surface motility with decreased *pilT* expression [215]. These findings taken together demonstrate BfmRS as a strongly supported point at which surface adhesins, capsule, envelope stress tolerance and resistance phenotypes are linked in *A. baumannii* [151,154,155,214,215].

### 5.2. Iron Limitation as a Shared Host Cue

Vertebrate hosts restrict iron availability and other nutrient metals as an innate defense against invasive bacteria [216]. In *A. baumannii* ATCC 19606^T^, acinetobactin synthesis and transport functions are not required for initial attachment to A549 alveolar epithelial cells; however, they are required for intracellular persistence, the induction of epithelial apoptosis, and for the significant killing of *G. mellonella* larvae and mice, establishing iron restriction as a condition met during host-infection rather than only in laboratory media [35,216,217]. This distinction raises the possibility that iron limitation serves as a positional cue that helps time the deployment of virulence factors, rather than only acting as a constraint on growth. Proper outer membrane localization of the acinetobactin receptor BauA depends on SecA (Section 2.1), such that loss of SecA function diminishes iron acquisition and decreases virulence in *G. mellonella* [23]. The two phospholipase Cs specific for phosphatidylcholine in ATCC 19606^T^ are iron regulated, linking membrane-damaging enzyme activity to the same cue [10]. Additionally, analysis of ATCC 19606^T^ under iron limitation showed increased CarO abundance and decreased OmpW abundance, indicating iron availability affects porin composition in opposite ways [104,105]. A strain producing severely truncated LOS similarly had deficient growth under iron-restricted conditions [120]. The two convergence points described here are themselves connected, with a Δ*bfmR* mutant showing increased expression of acinetobactin synthesis and transport genes, indicating BfmR may negatively impact this iron acquisition pathway [214]. Given BfmR’s positive effect on *csu* pili and capsule production, these transcriptomic observations suggest that envelope stress signaling and iron acquisition are linked, however not always aligned. These observations, predominantly derived from ATCC 19606^T^, indicate iron availability can act across all three virulence factor categories described in this review, influencing uptake systems, membrane permeability and secreted activity [10,23,105,120,214]. Whether these responses are coordinated, rather than independently acting on the same stimuli, has not been fully determined.

### 5.3. Outer Membrane Vesicles as a Shared Delivery Platform

While BfmRS and iron availability affect virulence at the level of regulation, OMVs integrate it at the level of physical packaging [8,47]. The biogenesis, protein content and immunological activity of *A. baumannii* OMVs are described in Section 2.3. OmpA, Omp33-36, LOS, and OXA-58, which requires SecA-dependent periplasmic translocation prior to vesicle release, all share the same export route [8,20,40,47]. Vesicle packaging therefore links factors that have no elucidated shared regulator, and interventions targeting OMV formation would be predicted to act on all of them simultaneously.

### 5.4. Recurring Features of the Proposed Coordinated Network

Three properties recur throughout the aforementioned three convergent points. The first is functional redundancy. PLC1 and PLC2 must be disrupted together before hemolytic activity and virulence are significantly reduced, all three PLDs must be removed before virulence is significantly reduced in vivo and in vitro, and Bap operates with BLP1 and BLP2 in biofilm formation and epithelial adherence [10,125,128]. Other examples include the recruitment of factor H being shared between several outer membrane proteins as well as their overlapping adhesion and invasion functions [49,88]. The second is the wide presence of trade-offs. Increased capsule production improves immune evasion and antibiotic resistance while reducing adhesion, biofilm formation and natural transformation [164,165,166]. Similarly, decreased OmpW under iron limitation decreases iron uptake capacity while decreasing colistin binding [103,104,105]. The same pattern is seen with loss of CarO increasing imipenem resistance while decreasing adhesion, invasion and dissemination [9,19]. The third is that some factors act directly on the host while others have a higher “position” and control whether those factors are produced or correctly positioned. Disruption of components like BfmRS and SecA affect many downstream functions, while others produce a narrower defect [23,155,214]. Together, these three properties are likely to complicate anti-virulence strategies. The first property makes it so inhibiting one member of a paralogous family may be insufficient [10,125]. Trade-offs mean suppressing one factor may potentially select for a more resistant phenotype, while the hierarchy of virulence factors means targeting a highly positioned target could inadvertently reintroduce the selective pressure of a conventional antibiotic that anti-virulence therapies are attempting to avoid [23,121,134,155]. Table 2 summarizes the anti-virulence targets discussed in this review.

### 5.5. Limitations of the Current Evidence Base

Much of the work elucidating the pathophysiology of *A. baumannii* is derived from a limited number of reference strains, particularly ATCC 19606^T^ and ATCC 17978, and connections which are inferred from transcriptomic data remain largely correlative. Coordinated regulation involving the three convergent points described above has not been demonstrated during human infection, and the extent to which this proposed framework holds across genetically diverse clinical isolates requires further investigation. This diversity has proven substantial across numerous pertinent virulence factors, with the *csu* cluster being prone to frequent gain and loss between isolates, Omp33-36 being highly polymorphic with strain-dependent transcription, and there being over 237 KL types reported [97,145,150,188]. Importantly, however, OmpA, CsuA/B, and Omp33-36 are each conserved in over 98% of strains examined, yet Omp33-36 was under-expressed in 76% of 100 MDR and XDR clinical isolates [54,92,95,96,187]. Similarly, the Bap epitope recognized by antibody 6E3 was only in 41% of outbreak isolates [127,136]. Virulence factor variation seems to occur due to differences at the level of genetic presence, sequence and expression, with conserved but conditionally expressed targets such as OmpW and Omp33-36 being encoded by nearly every isolate yet poorly presented during infection, further complicating the clinical effectiveness of a therapy.

Variation is also observed at the lineage level. A majority of MDR, XDR, and PDR isolates belong to two globally spread lineages, international clones I (IC I) and II (IC II), which correspond to sequence types (STs) 1 and 2 under the Pasteur multilocus sequence typing scheme, and carry resistance genes in distinct chromosomal resistance islands [218]. Biofilm formation and adherence to A549 cells was significantly higher for ST2, ST25 and ST78, yet *G. mellonella* killing was similar across genotypes [219]. IC I and IC II also differ in twitching motility, pellicle formation and capsular polysaccharide profile [220]. High-risk clones are therefore defined primarily by their resistance gene content, rather than distinct virulence phenotypes.

For these reasons, coordinated virulence is best regarded at present as an emerging conceptual framework. Establishing whether this coordinated network is a general attribute for *A. baumannii* will likely require validation across numerous clinical isolates, and therapies built on it will need to engage several virulence factors and be matched to isolates via rapid genomic testing.

## 6. Conclusions

**Figure 5:**
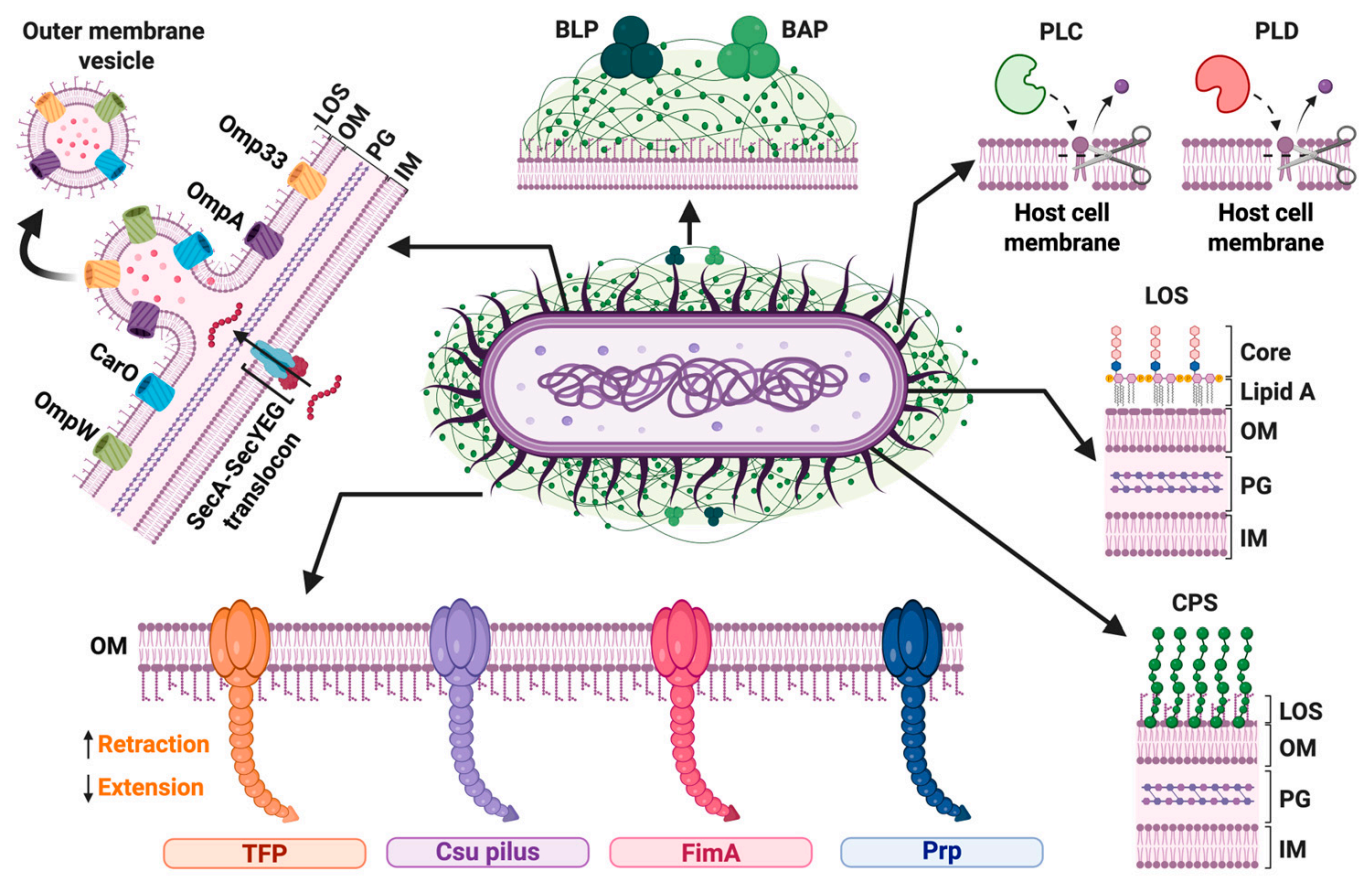
Schematic representation of major virulence factors of *A. baumannii*. This model illustrates the coordinated actions of surface structures, secreted factors and envelope components. Created in BioRender. Brancato, P. (2026) https://BioRender.com/gyga28t (accessed on 20 September 2026).

The pathogenicity of *A. baumannii* is driven by a diverse repertoire of virulence factors that collectively promote bacterial survival, contribute to host tissue damage and limit therapeutic efficacy [10,21,53,190] (Figure 5). Despite substantial progress in characterizing these individual factors, many of the mechanisms governing their function remain incompletely understood. In particular, a major gap in the field lies in our limited understanding of how these determinants are coordinated and regulated during infection, especially as *A. baumannii* transitions between environmental reservoirs and host-associated niches. Much of the existing knowledge is derived from studies examining single factors in a limited number of reference strains which may not fully capture the complexity of virulence as a dynamic and integrated system expressed in genetically diverse clinical isolates.

An emerging conceptual framework reframes virulence from a collection of discrete components towards a coordinated and interconnected network. Current evidence supports that many virulence traits are tightly linked through regulatory circuits and functional interactions between membrane structures, secreted factors, and environmental sensing pathways. Testing this framework will require the application of system-level approaches, including multi-omics, high-resolution infection models and computational network analyses, applied across diverse clinical isolates to define how virulence programs are orchestrated in space and time during infection.

Within this framework, the bacterial envelope has been proposed as a key hub of virulence coordination. OMPs, OMVs, LOS and CPS collectively form a multifunctional interface that contributes to immune evasion, antimicrobial resistance and host interaction. Rather than acting independently, these components may operate as a unified platform in which surface structures and regulatory signaling are closely coupled, although the extent of this coupling remains to be defined. Elucidating how these envelope-associated systems are coordinated and how they dynamically adapt to abiotic and host conditions represents a critical frontier in understanding *A. baumannii* pathogenesis.

Conceptual advances regarding these virulence factors directly inform new therapeutic strategies. Anti-virulence approaches, which aim to disrupt pathogenic mechanisms without directly killing the organism, offer a promising alternative to traditional antibiotics by potentially reducing selective pressure for resistance. Targeting processes such as biofilm formation, secretion systems or capsule biosynthesis may attenuate virulence while preserving commensal microbiota. To the extent that virulence systems are interconnected, effective interventions may require multi-target strategies that disrupt key nodes within these networks rather than individual factors in isolation. Identifying such vulnerabilities will depend on a deeper understanding of the regulatory hierarchies that govern virulence.

In parallel, phage-based therapies are gaining renewed attention as both treatment modalities and tools for probing bacterial biology. Bacteriophages can selectively target *A. baumannii*, and resistance to phage infection is often accompanied by alterations in surface structures such as the capsule or outer membrane proteins. These changes can reduce virulence or increase antibiotic susceptibility, highlighting opportunities to exploit evolutionary trade-offs between resistance and pathogenicity. Integrating phage therapy with conventional antibiotics and anti-virulence strategies may therefore enable synergistic and more durable treatment approaches.

Looking ahead, an emerging model describes *A. baumannii* virulence as a dynamic, adaptive system shaped by environmental cues, host interactions and evolutionary pressures. Continued advances in single-cell technologies, systems biology and infection modeling will be essential for capturing this complexity. Ultimately, testing whether virulence is best described as an integrated network and identifying the critical points of fragility within that network, may be key to developing next-generation strategies capable of overcoming *A. baumannii* infections caused by multidrug-resistant isolates. Direct evidence of coordinated regulation during human infection will be required before this framework can be regarded as an established feature of *A. baumannii* virulence and pathogenesis.

## Figures and Tables

**Table 1:** Overview of *A. baumannii* virulence factors reported in this review.

Category	Virulence Factor(s)	Function(s)	References
Membrane-associated virulence factors	SecA	Protein export, Omp localization, iron acquisition, carbapenemases translocation	[23,40,41]
	OmpA	Adhesion, invasion, apoptosis induction, serum resistance, biofilm formation, antibiotic permeability	[7,18,49,51,53]
	OMV	Shared delivery of OmpA, Omp33-36, LOS and carbapenemases, cytotoxicity, horizontal gene transfer	[8,20,40,47]
	Omp33-36	Adhesion, invasion, cytotoxicity, apoptosis induction, autophagy arrest, stress tolerance	[20,88]
	OmpW	Iron homeostasis, adhesion, invasion, biofilm formation, stress adaptation	[104,106]
	CarO	Carbapenem and ornithine transport, adhesion, invasion, dissemination	[9,19,21]
Outer envelope components and secreted virulence factors	LOS	TLR4-mediated inflammation, serum resistance, envelope integrity, colistin target	[121,134,137]
	Bap & Bap-like Proteins	Biofilm maturation, surface hydrophobicity, epithelial adherence	[127,128,140]
	Capsule	Immune evasion, complement and phagocytosis resistance, desiccation and disinfectant tolerance	[151,157,159]
	Phospholipases C & D	Membrane hydrolysis, cytolysis, epithelial invasion, serum resistance, iron acquisition	[10,124,125]
Pili and fimbriae	CUP	Biofilm initiation on abiotic surfaces, adhesion	[144,154,181]
	Type I pili & FimA	Adhesion under shear stress, pellicle and biofilm formation	[193,196]
	Type IV pili	Twitching motility, natural competence, adhesion, biofilm formation	[180,200]
	Light sensing, BlsA & Prp pili	Temperature-gated regulation of motility, biofilm formation and adhesion	[210,212,213]

Abbreviations: Omp, outer membrane protein; OMV, outer membrane vesicles; LOS, lipooligosaccharide; TLR4, toll-like receptor 4; Bap, biofilm-associated protein; CUP, chaperone–usher pili; Prp, photoregulated pilus.

**Table 2:** Summary of anti-virulence targets in *A. baumannii*.

Target	Proposed Strategy	Supporting Evidence	Limitations
SecA	Small-molecule inhibition of Sec-dependent export [42]	Required for BauA localization and virulence [23]; rose bengal blocks carbapenemase translocation and abolishes cross-protection [40,41]	Outer membrane permeation barrier [43]; dose-dependent reversal of effect [41]; broad role complicates dosing
OmpA	Active or passive immunization [54]	Highly conserved and immunogenic, with in vivo protection in murine models [54,55,56,57,58]; loss attenuates virulence and increases antibiotic susceptibility [18,49,52]	Serum resistance redundant across outer membrane proteins [49]; cytotoxicity partly OMV-delivered [8]
OMV	Whole-vesicle vaccination, including LOS-free preparations [85,87]	In vivo protection against pneumonia and sepsis [85,86]; retained by LOS-free vesicles [87]	High endotoxin in standard preparations [85]; composite target shared by several factors [8,20,40,47]; cargo control not fully elucidated
Omp33-36	Vaccine or antibody targeting of the Omp34 variant [92,94]	Highly conserved and immunoreactive, with in vivo protection [92,93,94]; deletion attenuates virulence [88]	Functional overlap with OmpA [88]; under-expressed in many MDR and XDR isolates and linked to imipenem resistance [95,96]; conservation disputed [97]
OmpW	Subunit or passive immunization [108]	Highly conserved, with in vivo protection and opsonophagocytic antisera [108]	Expression conditional on colistin resistance, iron and oxygen availability [103,105,106]; possible effect on colistin susceptibility unresolved [104]
CarO	Epitope source in computational vaccine designs [118,119]	Predicted antigenic and immunogenic in silico [118,119]; deletion attenuates virulence [9]	No in vivo protection data; loss associated with imipenem resistance [4,19,114]
LOS	LpxC inhibition [121]	In vivo protection attributed to prevention of a TLR4-mediated cytokine storm, without in vitro growth inhibition [121,137]	LOS loss associated with colistin resistance [121,134]; no in vitro activity to guide development [121]
Bap	Antibody interference with Bap [142]	Bap-deficient strains form immature biofilms [127,140]; antibody interference disrupts early biofilm [142]	Target epitope present in a minority of clinical isolates [127,140]; adherence redundant with Csu pili, OmpA and Bap-like proteins [128]
Capsule	Opsonizing antibodies or phage-derived depolymerases [167,168,169]	Opsonization enhances phagocytic killing and clearance [167]; depolymerases reduce serum survival and protect in vivo without selecting resistance [168,169]	Extensive K-type diversity restricts agents to matched types [145,150]; function sensitive to minor structural change [160]
Phospholipases	Miltefosine inhibition of phospholipase activity [175]	Inhibition reduces cytolytic activity and virulence [175]; combined *plc* deletion reduces hemolysis and virulence [10]; combined *pld* deletion increases colistin sensitivity [125,173]	Paralog redundancy, with attenuation requiring combined deletion [125]; substrate shared with eukaryotic membranes [10,125]
Csu pili	CsuA/B in a multivalent subunit vaccine [187]	Highly conserved, with partial in vivo protection improved by combination with FimA [187]	Cluster frequently gained and lost, with deleted isolates common at respiratory sites [188]; loss does not abolish adhesion [189]
FimA	FimA subunit or passive immunization, alone or multivalent [196,197]	Highly conserved [195]; with in vivo protection and reduced epithelial attachment [196,197,198]	Protection depends on antigen carriage and varies markedly between challenge strains [197]

Abbreviations: Omp, outer membrane protein; Bap, biofilm-associated protein; LOS, lipooligosaccharide; MDR, multidrug-resistant; OMV, outer membrane vesicle; TLR, toll-like receptor; XDR, extensively drug-resistant.

## Data Availability

All data generated or analyzed during this study are included in this published article.
